# Design of Radioiodinated Pharmaceuticals: Structural Features Affecting Metabolic Stability towards in Vivo Deiodination

**DOI:** 10.1002/ejoc.201601638

**Published:** 2017-04-26

**Authors:** Lorenzo Cavina, Dion van der Born, Peter H. M. Klaren, Martin C. Feiters, Otto C. Boerman, Floris P. J. T. Rutjes

**Affiliations:** ^1^ Institute of Molecules and MaterialsFaculty of ScienceRadboud UniversityHeyendaalseweg 1356525 AJ NijmegenNetherlands; ^2^ FutureChemistry Holding BV6525 ECNijmegenNetherlands; ^3^ Department of Animal Ecology & PhysiologyInstitute of Water & Wetland ResearchFaculty of ScienceRadboud UniversityPOB 90106500 GLNijmegenNetherlands; ^4^ Department of Radiology & Nuclear MedicineRadboud University Medical Center6500 HBNijmegenthe Netherlands

**Keywords:** Radiolabeling, Isotopic labeling, Radioiodinated pharmaceuticals, Iodine, Metabolism

## Abstract

Radioiodinated pharmaceuticals are convenient tracers for clinical and research investigations because of the relatively long half‐lives of radioactive iodine isotopes (i.e., ^123^I, ^124^I, and ^131^I) and the ease of their chemical insertion. Their application in radionuclide imaging and therapy may, however, be hampered by poor in vivo stability of the C–I bond. After an overview of the use of iodine in biology and nuclear medicine, we present here a survey of the catabolic pathways for iodinated xenobiotics, including their biodistribution, accumulation, and biostability. We summarize successful rational improvements in the biostability and conclude with general guidelines for the design of stable radioiodinated pharmaceuticals. It appears to be necessary to consider the whole molecule, rather than the radioiodinated fragment alone. Iodine radionuclides are generally retained in vivo on sp^2^ carbon atoms in iodoarenes and iodovinyl moieties, but not in iodinated heterocycles or on sp^3^ carbon atoms. Iodoarene substituents also have an influence, with increased in vivo deiodination in the cases of iodophenols and iodoanilines, whereas methoxylation and difluorination improve biostability.

## 1. Introduction

### 1.1. Iodine in Biology

Iodine is one of the heaviest essential elements present in living organisms. In biology, iodine differs from other halogens, because it is also stored in the form of organoiodides, whereas fluorine, bromine and chlorine are encountered mostly as salts or free anions^1^ Iodine is taken in through the diet in its inorganic iodide form and is absorbed in the gastrointestinal tract. Once in the bloodstream, iodide accumulates in tissues that express the selective sodium/iodide symporter (NIS). This symporter is expressed mostly in the salivary glands, gastric tissues, and thyroid gland.^2^ Iodide is secreted in the saliva; it has been suggested that this aids in maintaining the hygiene of the oral cavity and acts as a salvage mechanism to recycle iodide when the diet is deficient in iodine.^3^ In the stomach iodide is believed to have a role in regulating the gastric pH.^4^ Whereas it is taken up in the tissues mentioned above, there is also empirical evidence that iodide can actively be eliminated from the brain across the blood‐brain barrier (BBB) through a poorly understood mechanism.^5^

**Scheme 1:**
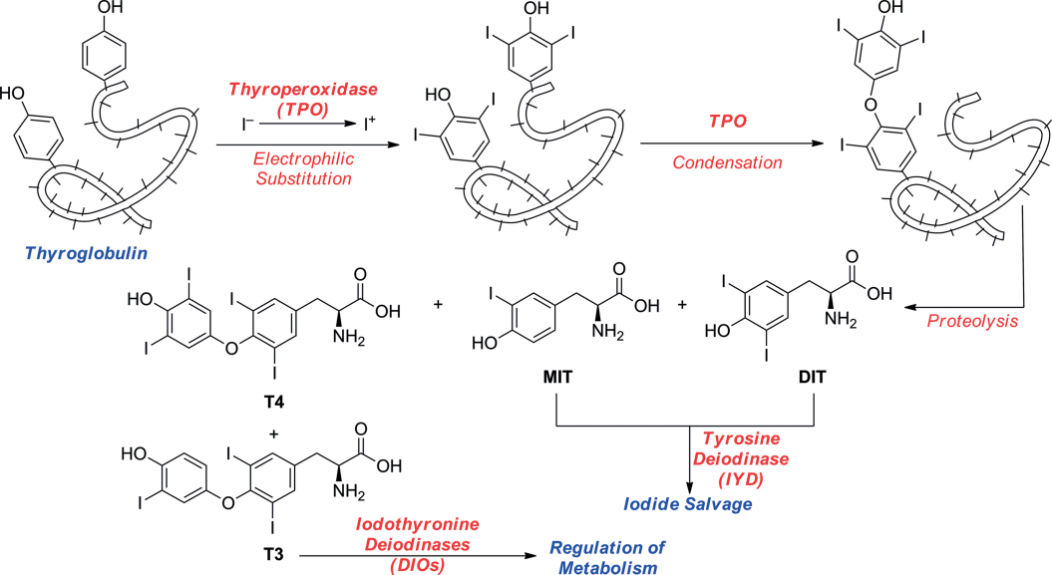
Biosynthesis of iodothyronines.

The thyroid gland is very efficient, much more so than the salivary glands and stomach, in clearing plasma of iodide. Iodide avidly accumulates to allow the synthesis of thyroid hormones (THs) T4 and T3 (Scheme 1). After uptake, through the action of the NIS, across the basolateral membrane of the thyrocyte, iodide is extruded across the apical membrane into the thyroid gland's follicle lumen. Here, iodide is oxidized to iodonium by thyroperoxidase (TPO) enzymes and mono‐ and disubstituted on the phenolic rings of the accessible fraction of the 137 tyrosine residues present in the glycoprotein thyroglobulin (a process known as “iodide organification”). Next, some of the iodinated phenolic rings (those with favorable spatial disposition; namely, hormonogenic residues) are coupled, with the terminal hydroxy group from one 3,5‐diiodotyrosine (DIT) residue attacking the *ipso*‐carbon atom either of another DIT residue or of a 3‐iodotyrosine (MIT) residue. After endocytosis of the luminal colloid contents, proteolysis of the oxidized thyroglobulin will result in the production of THs (a mixture of T4 and a small amount of T3) together with their intermediate unreacted byproducts MIT and DIT. Iodothyronines are then released into the bloodstream, where they travel through the body, mostly bound to carrier proteins.^6^ Specific selenocysteine‐dependent (Sec‐dependent) iodothyronine deiodinases (DIOs, enzymes reviewed in Section 2) activate/deactivate the THs by deiodination of the inner or the outer ring of the iodothyronine molecule.^7^

The synthesis of THs is a rather inefficient process: Sorimachi and co‐workers found that each oxidized thyroglobulin, after coupling between hormonogenic residues, affords – out of the native 137 tyrosine residues – an average of six molecules of MIT, six of DIT, three of T4, and one of T3.^8^ The MIT and DIT byproducts released after proteolysis are deiodinated by the flavin‐dependent iodotyrosine deiodinase (IYD, enzyme reviewed in Section 2), so the excess iodine and the tyrosine moieties can be salvaged.^9^

### 1.2. Iodine and the Brain

Other organs besides the thyroid gland can accumulate iodide, including the brain. In particular, the mammalian choroid plexus, a vascular network projecting into the brain's ventricles and secreting the cerebrospinal fluid (CSF), can actively take up and secrete iodide from and into the CSF.^10^ Moreover, the BBB, the highly selective epithelium that separates the brain from the circulating blood, is a major pathway for uptake of iodide into, and expulsion of iodide from, the brain.^11^ These and other early studies report that brain iodide transport is Na^+^‐dependent and perchlorate‐sensitive,[11b], 12 hinting at the involvement of the sodium iodide symporter (NIS). However, no NIS mRNA could be detected in human brain,^13^ except in breast cancer brain metastases.^14^ Similarly, the other thyroid iodide transporter, pendrin, could not be detected in human brain,^15^ leaving the exact iodide transport pathways in the brain still to be determined.

The efflux of iodide from the brain, by whatever pathway, has consequences for the development of radioiodinated pharmaceuticals. Okamura et al.[12b] mimicked the efflux of iodide liberated from a radioiodinated pentylpurine compound in mouse brain. Elimination of ^125^I from the brain was virtually complete after 20 min. The rapid loss of liberated iodide and the retention of a radiolabeled probe will most likely enhance the signal‐to‐noise ratio in, for instance, single‐photon emission computed tomography (SPECT) and positron emission tomography (PET) applications.

**Scheme 2:**
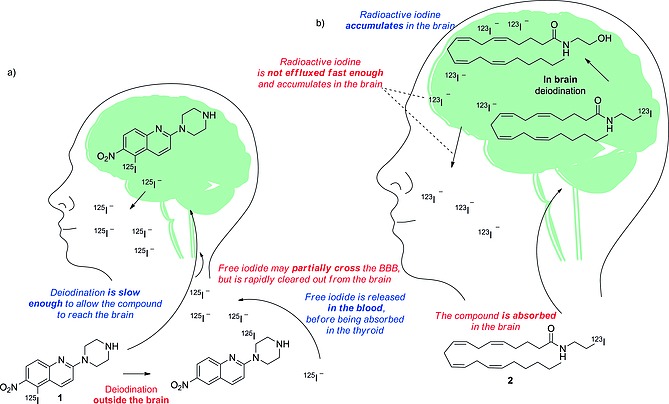
Schematic representation of the deiodinating metabolism of (a) compound **1**, and (b) compound **2**.

Despite the fact that compound **1** (Scheme 2) showed marked uptake of radioiodide in non‐target organs outside the brain (principally in the thyroid), it was successfully used to image the uptake of 5‐hydroxytryptamine in the brain.^16^ Presumably, uptake of compound **1** in the brain is faster than its deiodination. Compound **2** could not be used to image expression of cannabinoid receptors, due to nonspecific uptake in the brain.^17^ Presumably, the deiodination of compound **2** in the brain and the efflux of radioiodide from the brain is much slower than the deiodination of the tracer, resulting in accumulation of nonspecifically localized radioiodide in the brain (Scheme 2).

Strictly, in the development of a successful radioiodinated tracer for a target located in the brain, if the deiodination rate is slow enough to allow even a small proportion of the parent compound to reach its target, the peripheral (outside the BBB) deiodination might not affect the quality of the images. However, if the tracer is deiodinated in the brain, the iodide might be expelled too slowly, resulting in a low signal‐to‐noise ratio in the area of interest.

### 1.3. Iodine in Nuclear Medicine

A total of 37 isotopes of iodine are known, from ^108^I to ^144^I, of which ^127^I is the only stable nuclide. Of the radioactive isotopes, ^131^I, ^124^I, and ^123^I find broad application in nuclear medicine, whereas ^125^I is mostly used in biomedical research.

PET and SPECT are useful in vivo imaging techniques, allowing target areas to be imaged with millimeter resolution, by exploiting β^+^‐ and γ‐emission, respectively. In diagnostic nuclear medicine, a radioactive pharmaceutical (also called a tracer), designed to accumulate selectively in target tissue, is administered. The radioactive emissions of the tracer permit the distribution of the compound in the body to be visualized graphically. For instance, *meta*‐iodobenzylguanidine (MIBG), a widely used radioiodinated pharmaceutical in both therapeutic and diagnostic nuclear medicine, will accumulate selectively in tissues rich in adrenergic receptors (e.g., tumors such as pheochromocytomas and neuroblastomas). When using ^123^I‐MIBG, the γ‐emissions of ^123^I are visualized with a γ‐camera, allowing delineation of the tumor. In therapeutic nuclear medicine, the radioactive pharmaceutical will deliver the necessary radiation dose to the target tissue, allowing the destruction of the targeted tissue. For instance, when ^131^I‐MIBG is administered, the β^–^‐emissions of ^131^I will cause breaks in the DNA double strands and will destroy the tumor tissue. ^123^I is the most widely used iodine radionuclide for SPECT imaging because of the superior physical characteristics of its emissions (159 keV, *t*_1/2_ = 13.3 h). ^125^I is most often used in research, to perform in vitro testing in the development of radiopharmaceuticals or in displacement assays of a radioactive ligand. It has also been used to perform SPECT imaging, but – because its 35 keV emissions have a limited penetration range in tissues – it is only applied to small animals. ^131^I has a powerful β^–^‐emission (971 keV) that can be used for therapy and a 364 keV γ‐emission for imaging. ^124^I is a positron‐emitting radionuclide (β^+^ 603 keV) with a half‐life of 4.2 d. It is mainly used as an alternative to ^123^I for imaging studies with radioiodinated pharmaceuticals by PET instead of SPECT (Table 1).^18^

**Table 1:** Selected iodine isotopes used in nuclear medicine. EC = electron capture

Nuclide	*t*_1/2_	Decay	Main emission	Use in nuclear medicine
			[keV]	
^131^I	8 d	β ^–^	364	SPECT
		γ	971	therapy
^125^I	59 d	EC	35.5	research
^124^I	4.2 d	β^+^ and EC	603 (511)	PET
^123^I	13.22 h	EC	159	SPECT

The main drawback of radioiodinated pharmaceuticals is the low in vivo stability of the C–I bond. Dissociation of the radionuclide from the tracer results in unwanted accumulation of iodine radioactivity in the thyroid, stomach, and salivary glands. This will reduce the target‐to‐background ratio of the images. In therapeutic nuclear medicine, non‐target accumulation of iodine radioactivity will reduce the radiation dose to the target tissue and enhance the dose to non‐target tissues. Hence, in the design of radiopharmaceuticals, it is crucial to understand the metabolic transformations that they are likely to undergo in order to prevent or minimize deiodination.

### 1.4. Organic Iodides and Metabolic Transformations

Although much is known about the metabolism of endogenous organic iodides, far less is known about the metabolic fate of xenobiotics bearing this halide. Before analyzing the metabolic transformations that a radioiodinated pharmaceutical could undergo, it is important to understand the physicochemical properties of the C–I bond. The van der Waals radius of the iodine atom (as shown in Table 2) is comparable with that of a methyl group. It is substantially less electronegative than the other smaller halogens, with a value comparable with that of carbon.

**Table 2:** van der Waals radii^19^ and electronegativities^20^ for selected atoms/groups

	C	H	F	Cl	Br	I	CH_3_
van der Waals radius [Å]	1.70	1.20	1.35	1.80	1.95	2.15	2.00
Electronegativity	2.50	2.28	3.98	3.03	2.80	2.47	2.30

The C–I bond is weaker than those between carbon and the smaller halogens; this is considered to be due to the fact that the orbital overlap between carbon and iodine atoms is less efficient, resulting from the larger size of the iodine atom relative to that of the carbon atom. As can be seen in Table 3, the bond dissociation energy (BDE) varies according to the hybridization state of the carbon atom to which the iodine atom is attached: covalent binding to an sp^2^ carbon atom will result in a stronger interaction than that to an sp^3^ or sp carbon atom. In fact, the p orbital of the sp^2^ carbon atom can conjugate with the outer electron shells of the halogen atom, stabilizing the binding through resonance. Benzylic and allylic positions, however, show lower homolytic BDEs than other sp^3^ carbon atoms, because the radicals generated from the dissociation of the halide are stabilized by their structural and electronic features. Thus, the BDE is an important factor to be considered when designating the iodination site in the synthesis of a new radiopharmaceutical; it is, however, not the only important variable.

**Table 3:** BDEs^21^ calculated from Born–Haber thermodynamic cycles

R–I	Homolytic BDE	Heterolytic BDE
	[kJ mol^–1^]	[kJ mol^–1^]
CH_3_–I	234.72	889.52
C_2_H_5_–I	222.59	722.58
(CH_3_)_2_CH–I	222.17	628.48
(CH_3_)_3_C–I	210.87	562.33
CH_2_=CHCH_2_–I	184.51	671.95
CH≡C–I	206.27	748.10
C_6_H_5_CH_2_–I	167.36	566.93

**Scheme 3:**
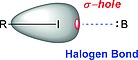
Schematic representation of the σ‐hole (red) of an organic (R = any hydrocarbon moiety) iodide and its interaction (halogen bond, blue dashed line) with a Lewis base (B).

**Scheme 4:**
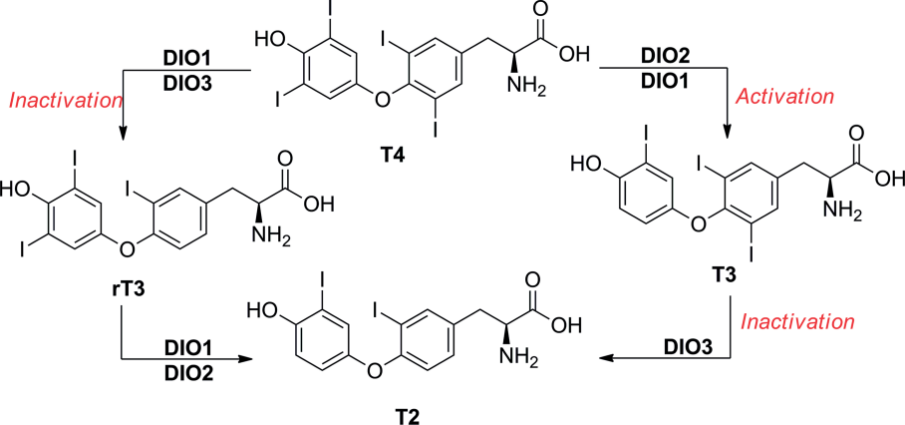
Inner‐ and outer‐ring dehalogenations catalyzed by the three DIOs.

**Scheme 5:**
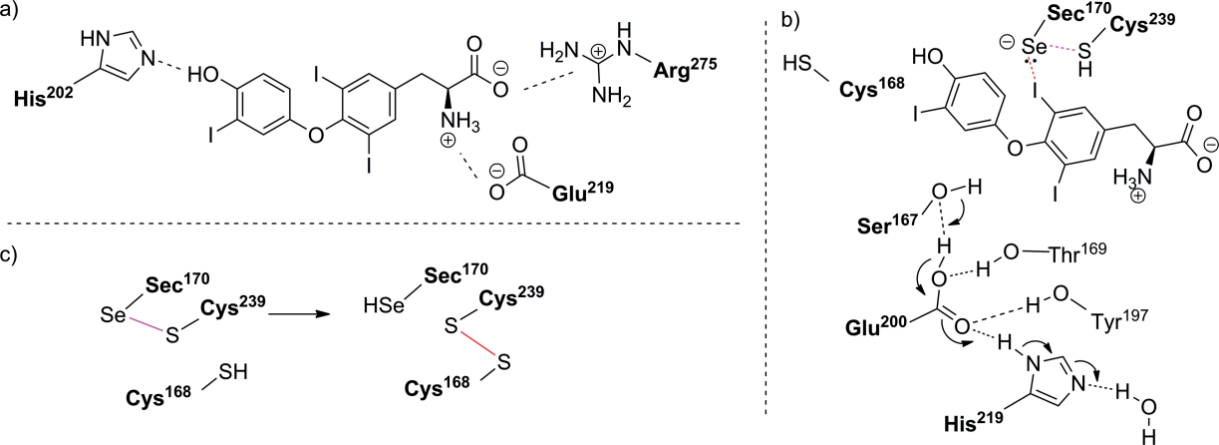
(a) Binding interactions between T3 and DIO3. (b) Reaction mechanism for IRD of DIO3. (c) Intramolecular recycling of the Sec residue, as proposed by Schweizer and co‐workers.^30^

It is important also to consider the concept of the σ‐hole, which is described as a positive region found in calculations of the electrostatic surface potential (ESP) areas of covalently bonded atoms of groups IV–VII. In the case of iodine, such a σ‐hole is located coaxially to the C–I bond (red in Scheme 3). The σ‐hole is the result of the anisotropy of the ESP of the halogen atom. This anisotropic model of the ESP was proposed in the last decade to explain the experimentally observed halogen bond (blue in Scheme 3) through a more coherent model than the London forces.^22^ The magnitude of the σ‐hole may affect the reactivity of organic halides: in fact, the halogen may form a halogen bond with a Lewis base in vivo, potentially weakening the C–I bond. An example of such an in vivo deiodination mechanism is the inner‐ring deiodination (IRD; Schemes 4 and 5b) of T4 or T3 by iodothyronine deiodinase 3 (DIO3), which is thoroughly reviewed in Subsection 2.2. The magnitude of the σ‐hole on the iodine atom may be exploited as a computed parameter to predict whether a given iodinated pharmaceutical will undergo in vivo deiodination. A higher magnitude of the σ‐hole will result in stronger halogen bonding with a Lewis base,^23^, ^24^ possibly weakening the C–I bond.

**Figure 1:**
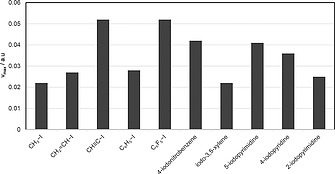
Magnitude [expressed in *v*_max_/a.u. (maximum magnitude/atomic unit)] of the σ‐hole on iodine in selected iodinated organic molecules, as calculated by Kolàř and co‐workers.^24^

**Figure 2:**
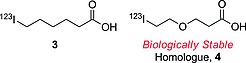
6‐Iodohexanoic acid and its biologically stable homologue.

Kolàř and co‐workers calculated the magnitudes for some simple organic iodides; their results are summarized in Figure 1.^24^ There is a slight increase in magnitude of the σ‐hole on iodine when bound to an sp^2^ (CH_2_CH–I and C_6_H_5_–I) rather than to an sp^3^ (CH_3_–I) carbon atom. A twofold increase is observed when iodine is bound to an sp (CH≡C–I) rather than to an sp^3^ (CH_3_–I) carbon atom. The magnitude of the σ‐hole on the iodine atom in an iodoarene is markedly affected by the nature and positions of the substituents. For instance, the magnitude of the σ‐hole on the iodine atom of iodobenzene (C_6_H_5_–I) is doubled upon substitution of its hydrogen atoms by the more electronegative fluorine atom (C_6_F_5_–I), it increases upon substitution with electron‐withdrawing groups (EWGs, e.g., nitro, CN, …) and decreases upon substitution with more strongly electron‐donating groups (EDGs, e.g., methyl, methoxy, …). The magnitude of the σ‐hole generally increases on iodine in iodoheteroaromatic systems, such as 5‐iodopyrimidine and 4‐iodopyridine, but, interestingly, decreases for 2‐iodopirimidine. For the design of radioiodinated pharmaceuticals it may be useful to calculate the magnitude of the σ‐hole in the chemical environment of interest in silico. As can be observed through this review, when radioiodine is installed on a more electron‐rich arene, the resulting iodinated radiopharmaceutical is observed to undergo less deiodination, so a value of 0.027 *v*_max_/a.u. (magnitude of the σ‐hole of iodobenzene) could be taken as a threshold as the maximum magnitude of the σ‐hole to prevent deiodination of an iodinated radiopharmaceutical. However, the composition and the structure of the whole molecule, rather than just of the radioiodination site, will affect the kinetics of the deiodination reaction: for instance, compound **3** (Figure 2) is known to be biologically not stable, whereas its homologue **4**, in which the methylene group at C4 is substituted with an oxygen atom, was found to be stable in vivo.^25^

To understand this behavior, it is necessary to understand the metabolic pathways a radioiodinated pharmaceutical might undergo. If the iodinated pharmaceutical of interest is known to be stable in an aqueous solution under standard conditions, it is likely that dehalogenation is a result either of some interaction with metabolic enzymes (deiodinases, formation of dehalogenating metabolites or nucleophilic enzymes) or of physical/chemical decomposition in vivo. In fact, organic iodides can often undergo physical/chemical decomposition. Thyroxine, for instance, is already known to deiodinate in vitro in aqueous solution, upon UV irradiation, or in a pH‐ and temperature‐dependent manner.^26^ Thus, when assessing the metabolic stability of an iodinated pharmaceutical, it is crucial not only to analyze the carbon–iodine BDE, but also to understand how the whole molecule might interact with any metabolic pathway in vivo.

In the next chapter, the enzymes that may catalyze deiodination are reviewed. Where possible, an in vitro test specific to each enzyme category is given; this may help to provide insight into the in vivo stability of a new radiopharmaceutical entity. Afterwards, the in vivo behavior of some selected radioiodinated pharmaceuticals is illustrated in terms of biodistribution data, obtained from previously published patents and peer‐reviewed literature. Finally, an attempt is made to correlate the metabolic dehalogenation rates of such pharmaceuticals with their structures and to determine, which structural features may hamper or favor metabolic deiodination.

## 2. Metabolic Pathways Leading to Deiodination

It is important to identify the enzymes and cofactors involved in the in vivo deiodination to estimate whether a new radiopharmaceutical entity, untested in vivo, will be biologically stable towards the loss of its iodine radionuclide. This section outlines the principal metabolic pathways that a radioiodinated pharmaceutical may undergo. After a discussion of the in vivo mechanisms of the different dehalogenation reactions (in terms of amino acids in the active sites of the enzymes involved or cofactors), some “enzyme‐free” testing methods for each specific pathway are presented.

### 2.1. Overview

Three main groups of enzymes that interact with radioiodinated pharmaceuticals can be identified:

1. Deiodinases, which specifically catalyze the reductive deiodination of an iodinated aromatic ring, often an *ortho*‐iodophenol.

2. Cytochrome P450 (CYP450) enzymes, which constitute the main class of enzymes responsible for the oxidation of xenobiotics in vertebrates.

3. Nucleophilic nonspecific enzymes, which may attack a particularly electrophilic carbon atom, as on a particularly strongly activated iodinated aromatic ring.

### 2.2. Deiodinases

Only two classes of mammalian deiodinases are known: iodothyronine deiodinases (DIOs) and iodotyrosine deiodinases (IYDs). Involved in the synthesis and regulation of thyroid hormones, they differ slightly in their target substrates (thyroid hormones and thyroid hormone byproducts, respectively), but drastically in their deiodination mechanisms.

#### Iodothyronine Deiodinases (DIOs)

DIOs are Sec‐dependent membrane proteins that catalyze the reductive elimination of iodide from iodothyronines (thyroid hormones). They are responsible for the activation and inactivation of thyroid hormone signaling (Scheme 4). T4 represents most of the circulating thyroid hormone and travels through the body bound to its specific protein carrier. It is a pro‐hormone, the activated form of which – T3 – binds to specific nuclear receptors that trigger signaling cascades leading to gene activation and consequent protein synthesis, accelerating cellular metabolism and other physiological processes, such as thermogenesis and lipolysis. rT3 and T2 are inactivated forms of the thyroid hormones, characterized by the loss of the iodine atom from the inner ring. DIOs regulate the homeostasis of the thyroid hormones, and they exist in three isoforms. They differ in kinetics, substrate, and location in the organism. The amino acid sequence in the active site is, however, highly conserved. Their principal characteristics are shown in Table 4.

**Table 4:** Characteristics of the different isoforms of DIOs

	Location	Substrate preference	Michaelis–Menten constant order
DIO1	liver, kidney, thyroid	rT3 (ORD) > T4 (IRD)	µm
DIO2	brain, placenta, thyroid, skeletal muscle, heart	T4 (ORD) > rT3 (ORD)	nm
DIO3	brain, placenta, pregnant uterus, fetal tissues	T3 (IRD) > T4 (IRD)	µm

**Figure 3:**
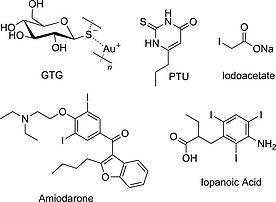
Selected known DIO inhibitors.

DIO1 catalyzes both outer‐ring deiodination (ORD) and inner‐ring deiodination (IRD), and it shows the highest affinity for rT3, in vitro, suggesting a scavenging role for this enzyme in the metabolism of thyroid hormones. Nevertheless, its activity is believed to contribute to a significant portion of the circulating T3. It is strongly inhibited by propylthiouracil (PTU; Figure 3), iodoacetate, and gold thioglucose (GTG). DIO2 is the main enzyme involved in thyroid hormone activation, catalyzing the ORD of T4 and generating T3. It is indeed responsible for most of the T3 produced in the brain. Amiodarone is a selective inhibitor for DIO2, but its pleiotropic effects and targets preclude its use for study of DIO2 in vivo.^27^ DIO3 has a protective role: through the inner‐ring deiodination (IRD) of T3 it terminates the thyroid hormone signaling and prevents its overactivation, especially during fetal growth. DIO2 and DIO3 are strongly inhibited only by GTG and iopanoic acid, which are nonselective DIO inhibitors.^28^, ^29^

**Figure 4:**
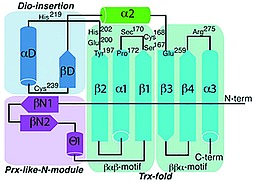
Schematic view of DIO3 tertiary structure. Catalytically important residues are shown. Reproduced from ref.^30^ (*Crystal structure of mammalian selenocysteine‐dependent iodothyronine deiodinase suggests a peroxiredoxin‐like catalytic mechanism*: U. Schweizer, C. Schlicker, D. Braun, J. Kohrle, C. Steegborn, *Proc. Natl. Acad. Sci. USA* **2014**, *111*, 10526–10531), with permission from the Proceedings of the National Academy of Science.

In 2014, the group of Schweizer and co‐workers^30^ solved the crystallographic structure of DIO3, unveiling its unique mechanism. Figure 4 shows a schematic view of its organization: it is a selenoprotein that shares most of its structure with the thioredoxin‐fold (Trx‐fold) protein (cyan in Figure 4). It also contains a peroxiredoxin‐like (Prx‐like) module (purple in Figure 4) and a lid (blue in Figure 4), which, by covering the substrate, is partially responsible for substrate recognition. Most of the catalytic residues are located in the Trx‐fold domain, on top of the βαβ‐motif. The main difference from the native Trx‐fold protein lies in the substitution in DIO3 of a cysteine residue by a Sec one. The protein is activated through homodimerization, displacing a phenylalanine residue that would instead occupy the catalytic cleft. His^202^, from the DIO‐insertion domain, acts as a binding partner, tethering the 4′‐phenolic end of the substrate, whereas the zwitterionic terminus interacts favorably with the carboxylate group of Glu^215^ and the guanidinum group of Arg^275^, allowing T3 to lie in the catalytic cleft (Scheme 5a). From the top side of the substrate, the selenide, formed by deprotonation of the Sec^170^ residue by a nearby His residue (not shown), behaves as a Lewis base and attacks the σ‐hole of the iodine, forming a halogen bond (Scheme 5b, red dashed line). The thiol group of Cys^239^ interacts with the selenium through chalcogen‐bonding (Scheme 5b, purple dashed line) to facilitate the cleavage of the C–I bond, inducing the transfer of electron density to its σ* orbital. The iodine atom is replaced with a proton approaching from the bottom side of the ring, conveyed by the triad of residues Ser^167^, Glu^200^, and His^219^ (Scheme 5b, blue arrow). This complex H‐bond network is further sustained by Tyr^197^ and Thr^168^. Although it was first believed that the nearby Cys^168^ residue was crucial in the catalytic reaction,^31^ the structure determined by Schweizer and co‐workers showed that in the reduced form of DIO3 this residue has its side chain pointing outwards from the catalytic cleft. When oxidized, DIO3 uses this thiol moiety to regenerate the selenyl residue. The selenol is restored by isomerization of the selenyl‐sulfide Sec^170^–Cys^239^ (purple in Scheme 5c) to the disulfide Cys^168^–Cys^239^ (red in Scheme 5c). The model of deiodination by DIO3 described above and shown in Scheme 5 is supported by theoretical density functional studies by Bayse and co‐workers,^32^ mechanistic studies by Manna and co‐workers,^33^ and the crystal structure by Schweizer and co‐workers.^30^

**Scheme 6:**
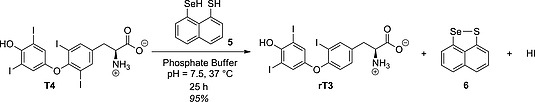
System engineered by Manna and co‐workers to mimic IRD.^31^

**Scheme 7:**
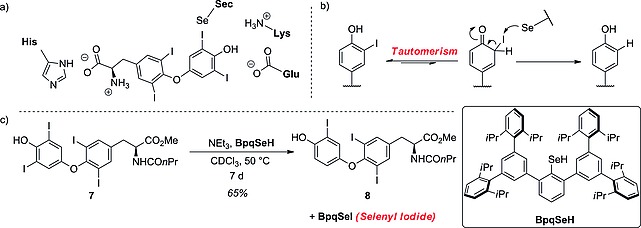
(a) Proposed docking of T4 in the DIO2 catalytic cleft (Callecaut and co‐workers).^34^ (b) Proposed reaction mechanism for DIO2 deiodination passing through a tautomeric intermediate. (c) Chemical system developed by Goto and co‐workers to mimic the Sec‐dependent dehalogenation.^35^

In trying to understand the catalytic mechanism of this enzyme, Manna and co‐workers^31^ developed a simple selenyl‐dependent chemical system, mimicking the reaction occurring at the catalytic cleft of DIO3 (Scheme 6). Compound **5** should act as the Sec^170^–Cys^239^ couple in DIO3, bearing both the selenol residue, to attack the iodine, and the proximal thiol group, to facilitate the cleavage of the C–I bond. In their experiments, they treated T4 with compound **5** under physiological conditions, achieving rT3 in high yield and recovering **6**, the oxidized form of **5**. This very simple assay, although it does not take the substrate selectivity of the enzymes into account, may be deployed to test the metabolic stability of an iodinated pharmaceutical towards IRD by DIO1 or DIO3 enzymes. Despite the bulk of experimental data, there is no coherent answer to the mechanism of ORD by DIO2. The homology of the catalytically relevant amino acids over the three types of DIO would seem to suggest a similar mechanism, as proposed by Schweizer and co‐workers. However, the replacement in the catalytic cleft of Arg^275^ for a lysine residue in DIO2 causes conformational changes in the binding site, which opened up the possibility that the substrate might lie in a reversed position (Scheme 7a). This docking position would still allow the Sec residue to adopt a favorable position to attack the iodine.^34^ It is unclear whether this mechanism proceeds via a tautomeric intermediate (Scheme 7b) or is induced by chalcogen bonding. While studying molecular cavities capable of stabilizing the selenyl iodide functionality, Goto et al. developed a reagent bearing a selenyl group capable of mimicking the reaction occurring at the Sec residue and also of stabilizing the formation of a BpqSeI adduct (Scheme 7c).^35^ In this dehalogenation process, a protected derivative of T4 – compound **7** – is heated in chloroform for 1 week, in the presence of BpqSeH, to yield the T3 homologue **8**. Although the conditions described by Goto are far from physiological, this reaction might be used in the future to develop methods to test the stability of a given iodinated pharmaceutical towards ORD by DIO2.

#### Iodotyrosine Deiodinase (IYD)

**Figure 5:**
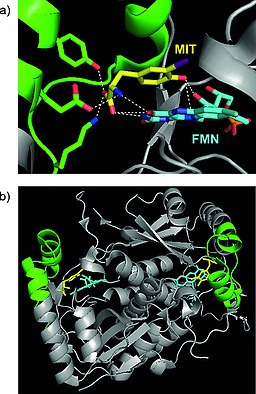
(a) Relevant interactions in the active site between IYD and MIT. Lateral chains from the lid interacting with MIT are highlighted in green, MIT in yellow, and the FMN prosthetic group in cyan. (b) Overall view of IYD with its substrate bound. The lid is highlighted in green, MIT in yellow, and the FMN prosthetic group in cyan. Adapted from Thomas et al. pdb ID: 3gfd.^9^

**Figure 6:**
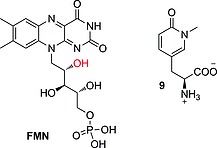
Flavin mononucleotide (FMN) and *N*‐methylpyrimidone.

IYD, found in the thyroid gland, is a flavin‐dependent oxidoreductase of the NADH oxidase/flavin reductase superfamily. It was crystallized in 2009 (Figure 5), bound to the reductive prosthetic group flavin mononucleotide (FMN; Figure 5 in cyan; see also Figure 6) and 3‐iodotyrosine (MIT).^9^ The active site is characterized by an α‐helix (green in Figure 5) constituting a lid that envelops the MIT substrate and the FMN group. The side chains from the lid are engaged in important binding interactions with the MIT substrate (Figure 5a), whereas the FMN is bound to more buried amino acids.

**Scheme 8:**
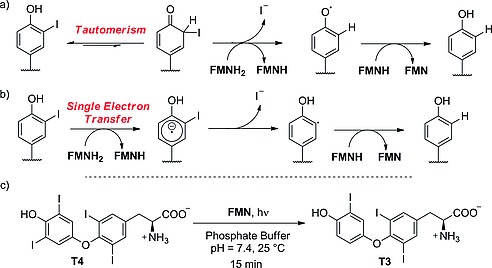
(a), (b) Proposed mechanisms for reductive dehalogenation by IYD (Rokita and co‐workers).^38^ (c) Non‐enzymatic deiodination as described by Reinwein and co‐workers.^39^

Binding between MIT and FMN is mediated by the 2′‐hydroxy group of FMN (red in Figure 6), which binds to the phenolic end of the substrate. This interaction is very important for substrate recognition; in fact, structure–activity relationship (SAR) studies conducted by McTamney and co‐workers revealed that substitution on the phenolic ring, increasing the p*K*_a_ of the phenol, resulted in improved binding.^36^ The same group also discovered that IYD is strongly inhibited by tyrosyl analogue **9** (Figure 6). Increasing or reducing the size of the *N*‐alkyl substituent resulted in reduced inhibition, showing a specific recognition site for an *ortho* substituent with the specific size of iodine.^37^ The mechanism of dehalogenation by IYD is quite unique in vertebrates, and only very recently, after having solved its crystal structure, did the group of Rokita propose two plausible possibilities (Scheme 8a and b).^38^ For the first, they proposed tautomerism as the driving force with FMN acting as a reducing agent (Scheme 8a). The second proposed mechanism (Scheme 8b) instead envisaged a single‐electron transfer to activate the iodinated phenolic ring, resulting in the reductive elimination of iodide. Both mechanisms have precedent in biochemistry, and further studies will be needed to understand the actual catalytic mechanism of this exotic enzyme. Reinwein's group described an in vitro deiodinating system involving T4 and FMN under physiological conditions (Scheme 11c, below).^39^ The substrate is slightly different from MIT or DIT; however, because the catalyzed reaction is similar (ORD of T4, in comparison with iodotyrosine deiodination), we would expect the mechanism to be similar too. In this system, FMN is added to a solution of T4 in phosphate buffer, while the reaction mixture is exposed to light. Total deiodination of T4 is usually observed in 15 min. This system may be deployed to test in vitro whether a new radioiodinated compound is stable or not towards IYD.

### 2.3. Oxidative Deiodination by CYP450

The cytochrome P450 (CYP450) family, expressed mostly in the liver and the lungs, represents the most important class of metabolic enzymes in aerobic organisms. It catalyzes the oxidation of xenobiotics in order to increase their hydrophilicity, thus facilitating their clearance from the blood and their consequent excretion in the urine as hydrophilic metabolites. The metabolic oxidation of iodinated hydrocarbons may lead to deiodination. In the following subsections we review the known oxidative deiodination of selected iodinated structural features. Finally, some considerations relating to the selectivity of CYP450 enzymes, which might be useful in the design of radioiodinated pharmaceuticals, are reported.

#### Iodoanilines

**Scheme 9:**
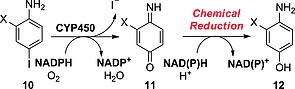
Reaction pathway proposed by Cnubben and co‐workers for the CYP450‐catalyzed dehalogenation of 4‐halogenated anilines. X = halogen atom.^40^

Cnubben's group described the concomitant deiodination of *para*‐iodoanilines such as **10** upon oxidation in the presence of CYP450 enzymes (Scheme 9).^40^ Oxidation to the aminoquinone derivative **11** by NADPH/O_2_ in the presence of CYP450 results in loss of iodide and insertion of an oxygen atom. Further reduction by NAD(P)H gives the deiodinated adduct **12**, in which the position previously occupied by iodine is oxidized to OH. This reaction has been observed to occur only in the case of *para*‐iodoaniline derivatives activated by a halogen in the *ortho* position to the amine group.

#### Aliphatic Iodides

**Scheme 10:**
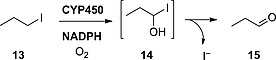
Deiodination mechanism based on hydroxylation as proposed by Tachizawa and co‐workers.^41^

Tachizawa and co‐workers proposed that CYP450 enzymes were responsible for the dehalogenation of terminal alkyl halides such as **13**. They observed that the most important reaction catalyzed by this enzyme family on substrates of this kind is the hydroxylation of the halogenated carbon atom (Scheme 10), resulting in a very unstable α‐hydroxy halide of type **14**, which would quickly lose the halogen, yielding the corresponding aldehyde **15**.^41^

#### Vinyl Iodides

**Scheme 11:**
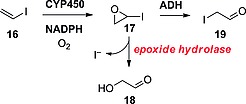
Oxidation of vinyl iodide as proposed by Guengerich and co‐workers.^42^

According to Guengerich and co‐workers, CYP450 would also oxidize vinyl iodides such as **16** to their corresponding epoxides **17** (Scheme 11).^42^ Epoxide hydrolase was then found to be responsible for the opening of the epoxide to glyoxaldehyde (**18**), with consequent loss of iodide. Alcohol dehydrogenase (ADH) would instead open the epoxide and oxidize the resulting iodoethanol derivative to iodoacetaldehyde (**19**), without loss of iodide.

#### Selectivity of CYP450 Enzymes

**Figure 7:**
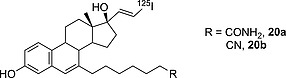
Estradiol analogues designed and synthesized by Neto and co‐workers.^43^

Despite the metabolic transformation described immediately above, a vinyl moiety is often chosen as a radioiodination site because of the high bond dissociation energy (BDE) of an sp^2^ carbon atom bound to an iodine atom (Table 3). The formation of deiodinating metabolites by CYP450 enzymes may be avoided by decreasing the lipophilicity of the whole molecule. For instance, Neto and co‐workers designed and synthesized the estradiol analogues **20a** and **20b**, differing in the nature of the R substituent (Figure 7).^43^ As can be seen from Table 5, compound **20b** (bearing a nitrile in place of an amide) is deiodinated much more rapidly than **20a**, with a very high residual radioactivity in the thyroid even 24 h after administration. To explain this result, it is necessary to understand how differently these two molecules will interact with their metabolic agent, CYP450. The main role of CYP450 enzymes is to catalyze the metabolism of xenobiotics to more polar (thus more hydrophilic) derivatives that can be excreted easily through the kidneys. Compound **20a** (bearing a terminal amide group) is more polar (C log P = 5.6 vs. 6.4) than **20b**, and then already more hydrophilic. Compound **20b**, being more hydrophobic than **20a**, needs to be further oxidized by CYP450 to be efficiently excreted, resulting in more loss of radionuclide.

**Table 5:** Radioactivity accumulation in rat thyroid as % ID/g organ and C log P for **20a** and **20b** (*n* = 4–5); ID = injected dose; C = computed; P = partition coefficient; *n* = number of experiments for each time point

	C log P	1 h	2 h	24 h	Ref.
**20a**	5.6	0.29 ± 0.07	0.49 ± 0.18	0.03 ± 0.01	43
**20b**	6.4	1.0 ± 0.2	0.9 ± 0.1	10.1 ± 3.1	43

**Figure 8:**
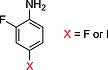
Compounds assayed by Cnubben against CYP450 oxidative dehalogenation.^40^ X = halogen.

With regard to the selectivity of CYP450, Cnubben et al. showed that increasing the size of the halogen (red in Figure 8) and the overall molecule results in reduced turnover rates of the enzyme (X = F, apparent *V*_max_ = 2.99; X = I, apparent *V*_max_ = 0.29).^40^ It appears that increasing the size of the molecule hampers its access to the CYP450 catalytic site.

The CYP450 isoforms differ drastically in terms of substrate selectivity and distribution in the body. The selectivity of CYP450 isoforms has already been reviewed in a recent work by Olsen and co‐workers and goes beyond the scope of this review.^44^

CYP450 enzymes may be inhibited by known CYP450 inhibitors such as isonicotinic acid hydrazide^45^ or fluconazole.^46^ It is important to mention that it is particularly difficult to mimic a metabolic CYP450 system. For instance, Cnubben et al. in their studies relied exclusively on in vitro testing, and their testing mostly involved proteins purified ex vivo because of the intrinsic difficulties of creating a CYP450 mimic system. Feiters and co‐workers reviewed the advances in obtaining CYP450 mimics; however, they all require very bulky and expensive reagents and often ad hoc supramolecular systems, required to simulate the complexity of this class of enzymes.^47^ The synthetic radiochemist, aiming to understand whether a radioiodinated pharmaceutical may be a substrate for CYP450 enzymes, may use a predicting software, in vitro incubation with tissue extracts, or animal models, as reviewed by Kirchmair and co‐workers.^48^

### 2.4. Nucleophilic Deiodination

In organic chemistry, iodine is also known to be an excellent leaving group in nucleophilic substitution reactions, so we would expect iodinated hydrocarbons to react with nucleophilic moieties, such as the thiol group in glutathione (GSH), resulting in conjugated adducts and release of iodide in the bloodstream. In addition, it is important to anticipate that some aryl iodides may react with GSH after previous intermediate oxidation by CYP450, as can be seen in the next subsections.^50^

#### para‐Iodonitrobenzenes/Iodoanilines

**Scheme 12:**
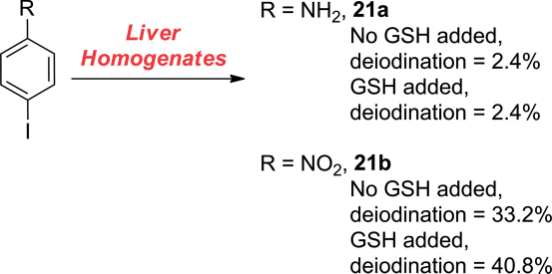
Results of the early studies by Sinsheimer and co‐workers.^49^

The first evidence of a GSH‐dependent dehalogenation, occurring in the human liver, was found by Sinsheimer and co‐workers.^49^ Assuming no deiodinases were implied in this process, Sinsheimer treated *para*‐iodoaniline (**21a**) or *para*‐iodonitrobenzene (**21b**) with liver homogenates either enriched in, or free of, GSH. He was able to observe that *para*‐iodoaniline (Scheme 12) was not significantly deiodinated, whereas *para*‐iodonitrobenzene could lose iodide, and that the rate of this metabolic process was accelerated by the addition of GSH. From his findings it is clear that an electron‐withdrawing group such as NO_2_ favors the conjugation of GSH, supporting a metabolic model in which GSH is conjugated by nucleophilic addition.

#### ortho‐Iodoanilines

**Scheme 13:**
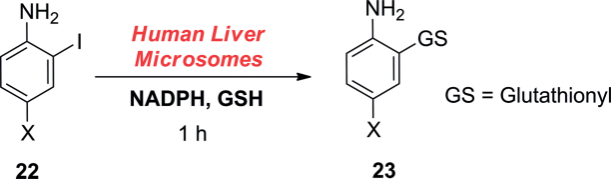
GSH‐dependent dehalogenation as described by Zhang and co‐workers.^51^ X = chlorine or bromine but not fluorine.

Zhang and co‐workers recently studied the metabolism of *ortho*‐iodoanilines and discovered that, after an initial oxidation, *ortho*‐iodoanilines of type **22** are conjugated with a GSH molecule, resulting in the 2‐deiodinated adducts **23** (Scheme 13).^51^ Although *para*‐iodoanilines are seemingly not deiodinated through a GSH‐dependent process (as seen in the previous subsection), *ortho*‐iodoanilines may undergo this metabolic transformation due to the proximity of the iodinated carbon atom to the reactive *ipso*‐carbon atom.

**Scheme 14:**
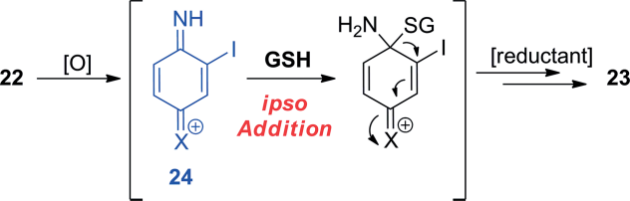
Mechanism proposed by Zhang and co‐workers.^51^

The actual mechanism of this metabolic reaction is rather complex, but Zhang and co‐workers proposed that it should proceed through the *ipso* addition of GSH to **24**, an oxidation product of intermediate **22** (blue in Scheme 14) formed upon initial oxidation by CYP450 enzymes. Next, the migration of the glutathionyl (GS) group to the 2‐position (concomitant with iodide elimination) would restore the aromaticity, yielding the dehalogenated adduct **23** conjugated to a glutathionyl molecule. This metabolic pathway is easily detectable because of the presence of the GSH conjugate. Because the biological reaction is complex, involving oxidation and GSH addition (at least two enzymes), it is difficult to define a chemical system capable of mimicking such a series of reactions, other than the use of liver homogenates. For the same reason, no selective inhibitor of this pathway can be defined.

### 2.5. Summary and Discussion

This section describes the metabolic pathways that may lead to deiodination. For each pathway, the enzymes and the cofactors involved are analyzed. When possible, some specific inhibitors for each enzyme are proposed. Furthermore, for dehalogenase enzymes, simple chemical systems able to replicate the reactions happening at the active site are described. However, those in vitro testing methods, although potentially able to avoid expensive in vivo assays, may be misleading. Thus, ex vivo testing is often preferred; as described by Owens and co‐workers, this consists of TLC analysis of blood extracts after administration of a non‐radiolabeled analogue.^52^ However, this latter form of test is only qualitative, so the most effective assay to determine the metabolic stability of a radioiodinated compound is the radio‐HPLC analysis of the ex vivo acetonitrile extracts of plasma samples at different time points, as further described by Owens and co‐workers.^52^

Lastly, now that the crystal structures of mammalian deiodinases have recently been determined, the radiochemist may perform docking studies of untested ligands on the crystal structures of the deiodinases, in order to predict whether a new radiopharmaceutical entity may be a substrate for such enzymes. Unfortunately, to date, no such studies have yet been published.

## 3. Structural Features Enhancing or Hampering the in Vivo Deiodination of Radioiodinated Pharmaceuticals

This section describes correlations between the structural features of radioiodinated pharmaceuticals and their radionuclide dissociation rates. In vivo deiodination has been assessed by analyzing biodistribution data, generally expressed as residual iodine radioactivity measured for each organ after euthanasia of the animal, reported in research journals and patents.

The capability of any metabolic pathway (such as those analyzed in the previous section) to cleave a radionuclide is strictly related to the whole structure. However, in the following subsections a tentative correlation of iodinated structural features (iodinated scaffolds common in medicinal chemistry) with their deiodination rates is proposed. Furthermore, some examples of rational drug design to avoid deiodination are given later in this review.

### 3.1. Carbocyclic Aromatic Moieties

#### Iodotyramines

**Figure 9:**
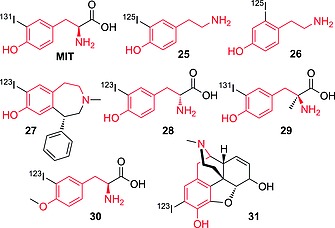
Selected compounds bearing radioiodine on a tyramine moiety (highlighted in red).

To obtain a stable tracer labeled with an iodine isotope, it is generally considered in medicinal chemistry that *ortho*‐phenolic positions should be avoided because of similarity with the outer ring of the thyroid hormones. In fact, there is good evidence of enzymatic in vivo deiodination occurring specifically on iodotyramine derivatives [i.e., derivatives of 4‐(2‐aminoethyl)‐2‐iodophenol (**25**), Figure 9]. The process is very likely enzyme‐catalyzed because of its intrinsic specificity: radiolabeled MIT and compounds **25**, **26**, and **27** are quickly deiodinated (MIT more rapidly than **27**),^53^, ^54^, ^55^ whereas even **28** (exogenous radioactive enantiomer of MIT) is instead metabolically stable.^56^ Minimal variations in the structure of MIT, such as α‐ or *O*‐methylation (compounds **29** or **30**, respectively) also result in almost complete retention of the radionuclide.^53^ Tyramine is the pharmacophore responsible for the binding of opiates to µ‐opioid receptors (MORs)^57^ and is present in morphine, the iodinated analogue **31** of which is reported to be metabolically stable towards deiodination.^58^ This behavior can be explained in terms of the selectivity of the deiodinase enzyme: compound **31**, being a complex natural product, has a strained three‐dimensional structure that likely does not fit the catalytic cleft of the deiodinating enzyme. On the other hand, compound **27** is metabolically not stable.^55^ Its tyramine moiety is embedded in a structure less strained than that in **31**, allowing the compound to adjust its three‐dimensional structure to fit the catalytic cleft of the deiodinating enzyme. Compounds **25** and **26**, being simpler than MIT (which is the endogenous substrate of iodotyrosine deiodinase, IYD), are both substrates for enzymatic deiodination, and show the minimal structure (iodotyramine) necessary for binding to the metabolic enzymes.

**Figure 10:**
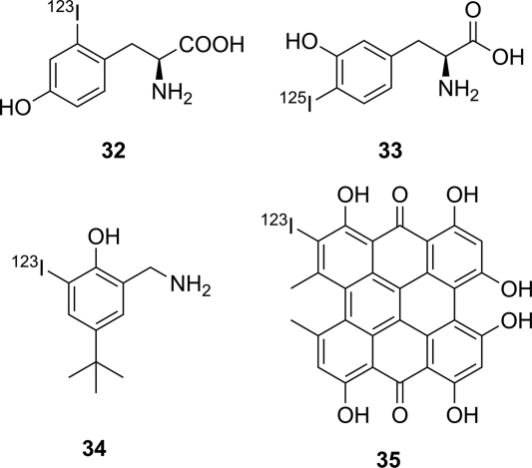
First row: selected compounds bearing radioiodine on a tyramine moiety. Second row: compounds bearing radioiodine on a phenol moiety.

Compound **32** (Figure 10) is only slightly deiodinated, with decreasing radioactivity accumulation in the thyroid, reaching a maximum value of 0.68 % after 15 min (ID/g, human sample, thyroid blocked). Compound **33**, up to 30 min p.i., is mostly excreted as the native compound (>96 %, urine), with only 4 % residual radioactivity resulting from free radioiodide.^59^ Compounds **32** and **33**, although only positional isomers of MIT, are more resistant to deiodination than the α‐methyl counterpart **29**. Analogue **29** may still be a substrate for deiodinases, whereas **32** and **33** are not substrates at all, demonstrating furthermore the selectivity of the enzyme(s) implicated in this particular deiodination process. Tyramine derivative **26** (Figure 9) is the decarboxylated analogue of **32**; its instability in relation to **32** may be due to the lack of one anchor point (i.e., ionizable moiety capable of strong electrostatic interaction with charged moieties in biomolecules) that would allow the molecule to fit better for its binding interactions within the binding cleft. It is difficult to determine which deiodinase [one of the iodothyronine deiodinases (DIOs) or iodotyrosine deiodinase (IYD)] is implied in this reductive deiodination process. However, given the higher similarity of iodotyramines to iodotyrosine than to iodothyronines, IYD may be the most likely enzyme leading to their deiodination. Furthermore, iodotyramines lack a second aromatic ring (present in thyroid hormones), and they may fit the catalytic cleft in DIOs less efficiently than in IYD.

#### Iodophenols

**Figure 11:**
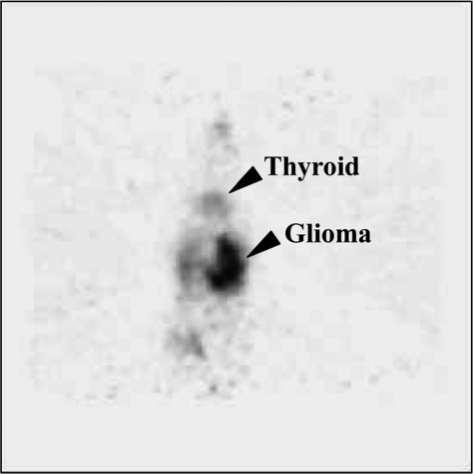
PET scan taken 1 h post injection (p.i.) of compound **35**. Reproduced from ref.^62^ (*Synthesis and in vitro/vivo Evaluation of Iodine‐123/124 Labelled Hypericin Derivatives*: S. W. Kim, J. H. Park, S. D. Yang, M. G. Hur, C. W. Choi, K. H. Yu, *Bull. Korean Chem. Soc*. **2008**, *29*, 2023–2025), with permission from the Korean Chemical Society.

Compounds **34** and **35** (Figure 10) are *ortho*‐iodophenols and are both assumed to be stable towards deiodination, showing a very mild accumulation of iodine radioactivity in the thyroid (for **34**)^60^ and in the stomach (only relevant data given for **35**)^61^ as shown in Table 6. However, further analysis performed with **35** (Figure 11)^62^ revealed that some deiodination could occur, with a PET scan of an injected mouse showing some uptake in the thyroid.

**Table 6:** Accumulation of iodine radioactivity in selected organs for compounds **34**–**36**

	Organ	2 min	10 min	15 min	0.5 h	1 h	4 h	6 h	24 h	Ref.
**34**	stomach^a^	n.a.	n.a.	2.57 ± 1.92	2.38 ± 0.88	2.33 ± 0.17	1.82 ± 0.4	1.13 ± 0.27	0.12 ± 0.08	60
	thyroid^a^	n.a.	n.a.	0.02 ± 0.01	0.02 ± 0	0.02 ± 0	0.05 ± 0.02	0.11 ± 0.05	0.41 ± 0.22	60
**35**	stomach^b^	n.a.	0.4 ± 0	n.a.	n.a.	n.a.	0.9 ± 0.2	n.a.	0.8 ± 0.2	61
**36**	thyroid^c^	0.156 ± 0.005	n.a.	0.064 ±0.005	0.073 ± 0.015	0.087 ± 0.007	0.376 ± 0.016	n.a.	2.32 ± 0.25	63

a ID/g %, rats, *n* = 10.

b ID/organ %, mice, *n* = 3.

c ID/organ %, rats, *n* = 3.

Rather than being enzymatically deiodinated, **35** is more likely to lose the radionuclide due to secondary reactions with oxidizing/reducing agents present in tissues because of its very dense and bulky carbon core, which is not very likely to fit into the active site of an enzyme evolved to fit an iodotyrosine or an iodothyronine. Also, the iodinated ring is much more activated than in **34**, with **35** being a planar, aromatic system made up of eight unsaturated six‐membered rings, thus making the C–I bond more labile. Although not directly due to deiodinases, the mild deiodination of iodohypericyn **35** might be a result of tautomerism, which would facilitate nucleophilic attack on the iodide by any thiol moiety.

**Figure 12:**
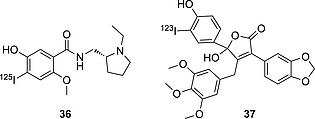
Selected compounds bearing radioiodine on an *ortho*‐iodophenol moiety.

Compound **36** (Figure 12) is biologically incredibly stable,^63^ most likely due to the different positions of the OH and I groups in relation to those in a native iodotyramine. Also, the halogenated aromatic ring contains a methoxy group, which, by donating electron density through resonance, is likely to stabilize the C–I bond.

On the other hand, the endothelin receptor imaging agent **37** could not be applied successfully because of nonspecific accumulation in the liver and in the plasma.^64^ Although no metabolic data are given, nor data relating to any eventual accumulation in the thyroid, this result likely reflects two metabolic processes: glucorinidation (accounting for the accumulation in the liver) and deiodination (likely resulting in high plasma accumulation). In addition, the metabolites produced by the fragmentation of compound **37** may become good substrates for deiodinase enzymes.

#### Iodoanilines

**Figure 13:**
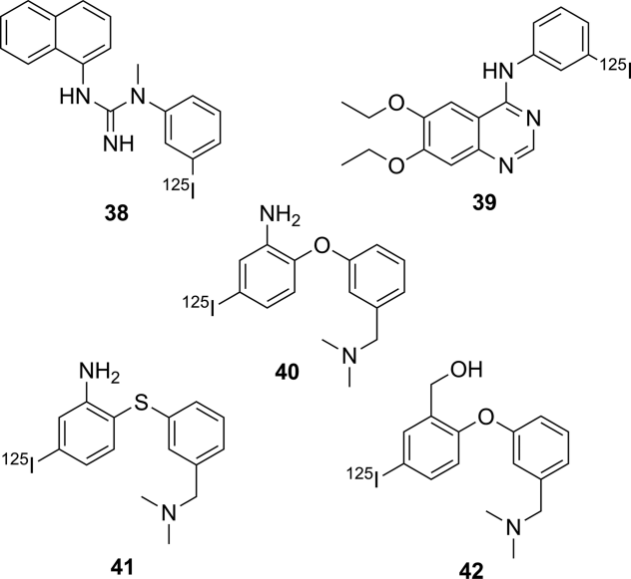
Selected compounds bearing radioiodine on an aniline derivative moiety.

Aniline is a common scaffold in medicinal chemistry. It is often chosen for radioiodination, being particularly activated towards electrophilic substitution. However, as was shown in the previous section, iodoanilines are a class of substrate for CYP450 metabolic transformations (especially *para*‐iodoanilines) and/or GSH addition (especially *ortho*‐iodoanilines), resulting in loss of the radionuclide. Most pharmaceuticals incorporating iodoaniline have the iodo substituent in the *meta* position (Figure 13). Nevertheless, this particular molecular scaffold is always prone to deiodination. This behavior may be explained in terms of the capability of the aniline moiety to be partially protonated in vivo: the positively charged substituent on the aromatic ring will decrease the C–I bond dissociation energy (BDE) and widen the σ‐hole on the iodine, making the molecule more prone to deiodination. In fact, in vivo pharmacokinetic studies of compound **38** (Figure 13), bearing a guanidine substituent (largely protonated under physiological conditions), showed the radionuclide to be completely lost in ca. 5 min p.i. (*t*_1/2_ = 2.17 min).^52^ Compound **39** (Figure 13) is more stable than **38**, but it is still significantly deiodinated in vivo, already showing a very low target‐to‐background signal at 1 h p.i., as found by Hirata and co‐workers.^65^ Although the biodistribution data (Table 7) for compound **39** cannot be directly compared with those for **40**–**42** (Figure 13),^66^, ^67^, ^68^ because no thyroid data are given for **39** and the experiments were conducted on different species, there is evidence for compound **40** being less prone to deiodination than **39**. Interestingly, adding an electron‐donating substituent (O/SAr in **40** and **41**) to the *meta*‐iodoaniline moiety results in increased biostability of the scaffold. Moreover, the replacement of the oxygen atom in **40** with sulfur in **41** leads to a more stable analogue. In fact, the outer electrons of sulfur, because of its reduced electronegativity relative to oxygen, are more available to be donated through resonance, making the substituent even more electron‐donating. Most likely, electron‐donating substituents increase the biostability of iodoanilines. Interestingly, compound **42**, not containing an aniline moiety, with the nitrogen substituted with a CH_2_OH group, proved to be metabolically completely stable, showing zero accumulation of iodine radioactivity in the thyroid. This comparative result would further support the hypothesis that anilines are not a biologically particularly stable group for radioiodination.

**Table 7:** Accumulation of iodine radioactivity for compounds **39**–**42**

	5 min	15 min	0.5 h	1 h	2 h	4 h	6 h	12 h	24 h	Ref.
**39** a	2.79 ± 0.59	16.04 ± 1.86	21.18 ± 4.05	28.62 ± 5.33	16.31 ± 2.10	n.a.	15.28 ± 2.48	n.a.	n.a.	65
**40** b	0.10 ± 0.02	n.a.	0.15 ± 0.04	0.41 ± 0.11	0.96 ± 0.06	2.34 ± 0.06	2.68 ± 0.66	5.15 ± 1.49	4.61 ± 1.21	66
**41** c	0.08 ± 0.03	n.a.	0.13 ± 0.002	0.22 ± 0.08	0.63 ± 0.20	1.28 ± 0.67	1.31 ± 0.21	n.a.	n.a.	67
**42** d	0.08 ± 0.02	n.a.	0.04 ± 0.01	0.03 ± 0.01	0.03 ± 0.01	0.04 ± 0.04	n.a.	n.a.	n.a.	68

a Stomach, ID/g %, mice, *n* = 4.

b Thyroid, ID/organ %, rats, *n* = 3.

c Thyroid, ID/organ %, rats, *n* = 3.

d Thyroid, ID/organ %, rats, *n* = 3.

#### Anilines – Drug Design Strategy Example

**Figure 14:**
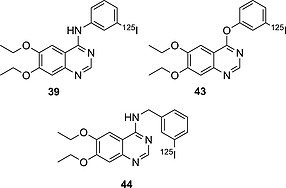
EGFR TK imaging agent candidates.

In their efforts to obtain radioiodinated EGFR‐TK imaging agents, Hirata and co‐workers synthesized the analogues depicted in Figure 14 69 After they observed that the first analogue they obtained – **39** – deiodinated rapidly in vivo (see Table 8; the high accumulation of iodine radioactivity in the stomach already after 15 min p.i.), they proceeded by trying to modify the spacer between the quinazoline ring and the iodinated moiety. Firstly, from an iodoaniline they switched to an iodophenoxy moiety, yielding compound **43**, which displayed a more than fourfold increase in biostability. In another analogue – **44** – they inserted a one‐carbon spacer between the nitrogen atom and the aromatic iodinated moiety, giving a compound twice as biostable as **43**. In vivo protonation of the quinazoline ring is likely to activate the iodoaromatic moiety towards dehalogenating metabolism. This effect is more pronounced when the linker is a nitrogen atom than when it is a carbon or oxygen atom.

**Table 8:** Accumulation of iodine radioactivity in the stomach (mice) for compounds **39**, **43**, and **44**. Data are given as ID/g %, *n* = 3

	5 min	15 min	0.5 h	1 h	2 h	3 h	6 h	Ref.
**39**	2.79 ± 0.59	16.04 ± 1.86	21.18 ± 4.05	28.62 ± 5.33	16.31 ± 2.10	19.85 ± 2.80	15.28 ± 2.48	69
**43**	2.08 ± 0.43	7.88 ± 2.38	5.06 ± 2.59	3.56 ± 0.89	6.85 ± 0.13	5.20 ± 1.05	8.39 ± 1.13	69
**44**	1.60 ± 0.27	4.00 ± 0.64	4.70 ± 0.60	3.05 ± 0.72	3.65 ± 1.14	3.71 ± 0.54	3.89 ± 1.03	69

#### Iodoarenes

In medicinal chemistry, it is commonly assumed that aryl moieties are good sites for radioiodination,^73^ firstly due to the ease of attachment of the radionuclide to such fragments, and secondly due to the high energy of the thus‐formed Ar–I bond. In the following subsection some successful radioiodinated pharmaceuticals bearing the radionuclide on simple arenes are reviewed and compared with other compounds radioiodinated in different positions as discussed in previous subsections. Next, it is discussed how the relative position of iodine (*ortho/meta/para*) and different substituents may affect the deiodination rates.

**Figure 15:**
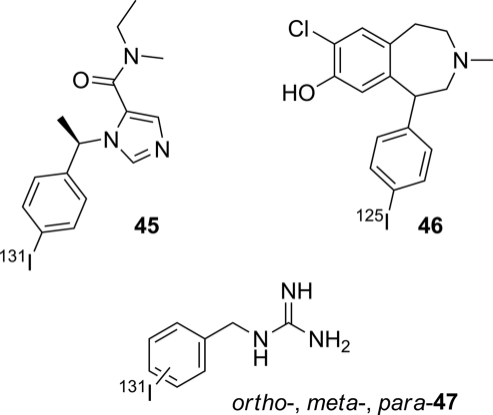
Selected compounds bearing radioiodine on an aryl moiety.

Compound **45** (Figure 15), patented by the Julius Maximilians University of Würzburg, was stable in mice, displaying accumulation of iodine radioactivity below 5 % (ID/g) until 4 h p.i.,^74^ thus demonstrating that iodoarenes are biologically generally stable. Compound **46** is an analogue very similar to compound **27** (Figure 9), in which the radionuclide was shifted to the non‐phenolic, aromatic moiety. This modification resulted in greatly improved biostability, as can be seen in Table 9, with almost no significant accumulation of iodine radioactivity being observed in the thyroids of rats treated with compound **46**.^71^

**Table 9:** Accumulation of iodine radioactivity in the thyroid for compounds **27**, **46**, and the isomers of compound **47**

	2 min	15 min	0.5 h	1 h	2 h	3 h	24 h	48 h	72 h	Ref.
**27** a	n.a.	n.a.	n.a.	3.2 ± 0.98	n.a.	14 ± 2	19 ± 3	23 ± 6	n.a.	70
**46** b	0.09 ± 0	0.04 ± 0.01	0.03 ± 0.01	0.03 ± 0	n.a.	n.a.	n.a.	n.a.	n.a.	71
*para*‐**47** c	0.5 (0.3–1.0)	n.a.	n.a.	n.a.	0.9 (0.6–1.0)	n.a.	9.0 (4.3–7.3)	13.6 (3.7–27.4)	33.6 (27.4–42.4)	72
*ortho*‐**47** c	0.3 (0.2–0.3)	n.a.	n.a.	n.a.	0.4 (0.2–0.3)	n.a.	2.6 (1.2–4.0)	9.4 (9.0–9.8)	n.a.	72
*meta*‐**47** c	0.6 (0.5–0.7)	n.a.	n.a.	n.a.	0.6 (0.5–0.6)	n.a.	3.4 (2.1–4.7)	1.3 (0.5–2.1)	n.a.	72

a ID/organ %, rats, *n* = 4.

b ID/organ %, rats, *n* = 4, 6, 5, 3.

c ID/g %, dogs, *n* = 3.

*meta*‐Iodobenzylguanidine (MIBG) – compound *meta*‐**47** (Figure 15) – is a widely used radioiodinated pharmaceutical in nuclear medicine, both in therapy and diagnosis. While performing lead optimization studies, aiming to find a powerful imaging and therapeutic agent based on an iodobenzylguanidine scaffold, Wieland and co‐workers synthesized the *ortho/meta/para* isomers of compound **47** and studied their biodistribution profile in mice.^72^ The accumulation of iodine radioactivity in the thyroid (shown in Table 9) shows the *para* isomer to be the fastest in dissociating from the radionuclide, whereas deiodination is not an important metabolic pathway for the *meta* isomer. The *ortho* isomer displays intermediate behavior.

**Scheme 15:**
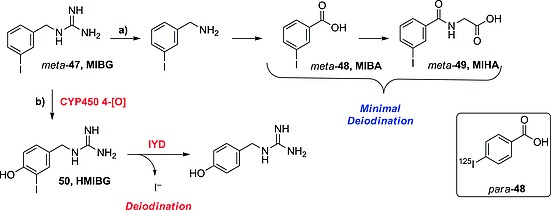
In vivo metabolism of MIBG (*meta*‐**47**) as proposed by Rutgers^76^ and Wafelman.^75^ Rectangle: *para* isomer of MIBA, studied in vivo by Garg et al.^77^

To explain this result it is important to understand the metabolism of iodobenzylguanidines. The groups of Wafelman^75^ and Rutgers^76^ studied the metabolites formed after administration of *meta*‐**47** (Scheme 15). Although the molecule was mainly excreted intact (70–85 %), a small fraction was found to undergo non‐aromatic oxidative metabolism (Scheme 15a) to give mostly *meta*‐iodobenzoic acid (MIBA, *meta*‐**48**) and *meta*‐iodohippuric acid (MIHA, **49**),^75^ which were minimally deiodinated (see Table 10 for thyroid accumulation data for *meta*‐**48** studied alone)^77^ In a very few patients they observed trace amounts of aromatic oxidation (Scheme 15b), yielding the minor metabolite 4‐hydroxy‐3‐iodobenzylguanidine (HMIBG, **50**), which was quickly deiodinated. The deiodination is likely performed by IYD, metabolite **50** being structurally similar to an iodotyramine. Aromatic oxidation of the *para* isomer is likely to result directly in loss of the radionuclide, explaining the faster accumulation of iodine radioactivity in the thyroid. In addition, compound *para*‐**47** will also form metabolites analogous to those formed by *meta*‐**47** through the pathway depicted in Scheme 15a, in the form of *para*‐iodohippuric acid and *para*‐benzoic acid (compound *para*‐**48**, shown in Scheme 15), which are more rapidly deiodinated than their *meta* isomers, as shown in Table 10.

**Table 10:** Accumulation of iodine radioactivity in the thyroid (mice) for compounds *para*‐ and *meta*‐**48**, expressed as ID/g %, *n* = 5, as reported by Garg and co‐workers^77^

	1 min	2 h	4 h	6 h
*para*‐**48**	0.17 ± 0.04	0.20 ± 0.05	0.33 ± 0.09	0.38 ± 0.06
*meta‐* **48**	0.13 ± 0.03	0.14 ± 0.03	0.23 ± 0.07	0.28 ± 0.04

Garg and co‐workers studied the radionuclide dissociation of the constitutional isomers of iodobenzoic acid (IBA; Scheme 15, compounds *meta*‐ and *para*‐**48**), and stated that its *para* isomer is 1.5 times (until 6 h) more prone to deiodination than the *meta* isomer (Table 10).^77^ Their result is consistent with that obtained before, when comparing the accumulation of iodine radioactivity in the thyroid at 6 h p.i. of the *meta* and *para* isomers of **47**. Deiodination is less important for the isomers of **48** than for those of **47**, because the metabolic products of IBA are not likely to be further oxidized, so they may not be substrates for IYD.

#### Substituent Effects – Electron‐Withdrawing versus Electron‐Donating Groups

**Figure 16:**
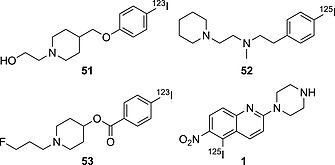
Selected compounds bearing radioiodine on the aromatic moiety, from EDG (left) to EWG (right) substituents.

As anticipated before, it is likely that electron‐donating substituents are able to stabilize C–I bonds in iodoarenes in vivo. The compounds in Figure 16 form a series from more electron‐donating to electron‐withdrawing substituents on their aromatic ring, and their biostability (Table 11) decreases with increasing electron‐withdrawing character of the substituent.

**Table 11:** Accumulation of iodine radioactivity in the thyroid for compounds **51**, **52**, **53**, and **1**

	5 min	1 h	2 h	4 h	6 h	8 h	12 h	16 h	24 h	48 h	Ref.
**51** a	n.a.	0.08 ± 0.01	n.a.	0.08 ± 0.02	n.a.	0.09 ± 0.04	n.a.	n.a.	0.10 ± 0.04	0.13 ± 0.06	78
**52** b	n.a.	0.19 ± 0.02	n.a.	n.a.	0.20 ± 0.05	n.a.	n.a.	n.a.	0.58 ± 0.03	n.a.	79
**53** a	n.a.	0.94 ± 0.37	n.a.	1.34 ± 1.15	1.33 ± 0.56	n.a.	n.a.	0.93 ± 0.45	2.97 ± 0.44	n.a.	78
**1** c	0.19 ± 0.15	n.a.	4.7 ± 2.4	n.a.	11 ± 1	n.a.	14 ± 2	n.a.	n.a.	n.a.	16

a ID/organ %, mice, *n* = 5.

b ID/organ %, rats, *n* = 4.

c ID/organ %, rats, *n* = 3.

Compound **51**, in which the iodoarene moiety is linked to an ether (EDG) is the biologically most stable (max ID/g % thyroid = 0.13).^78^ Shifting to a less strongly electron‐donating substituent (–CH_2_CH_2_NR_2_), as in compound **52**, results in more in vivo dehalogenation (max ID/g % thyroid = 0.58).^79^ Furthermore, switching to an electron‐withdrawing substituent (–COOR, compound **53**) drastically increases the rate of deiodination (max ID/g % thyroid = 2.97).^78^ Compound **1**, in which the iodoaromatic moiety is embedded in a quinolinic scaffold (capable of being protonated in vivo) and also bears a very strongly EWG (–NO_2_), is very quickly deiodinated in vivo, already displaying a very high uptake of iodine radioactivity in the thyroid at 2.0 h p.i.^16^

#### DiMagno Method for Hampering Deiodination

**Figure 17:**
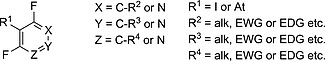
Structures stabilized by vicinal difluorination.

DiMagno, of Ground Fluor Pharmaceuticals Inc., recently patented a method to reduce in vivo loss of radionuclide of iodinated or astatinated aromatic pharmaceuticals.^80^ His rationale is based on vicinal difluorination (around the radionuclide, Figure 17), which, according to his findings, results in a higher BDE of the carbon–radionuclide bond. Fluorine, despite being a very electronegative halogen, has several unbound electron pairs that are likely to be donated to the aromatic ring through resonance, making the carbon–iodine bond stronger. In addition, vicinal fluorination does not allow hydroxylation by CYP450, avoiding the formation of metabolites that may undergo deiodination through tautomerism or enzymatic deiodination by deiodinases.

### 3.2. Heterocycles

#### Indoles

**Scheme 16:**
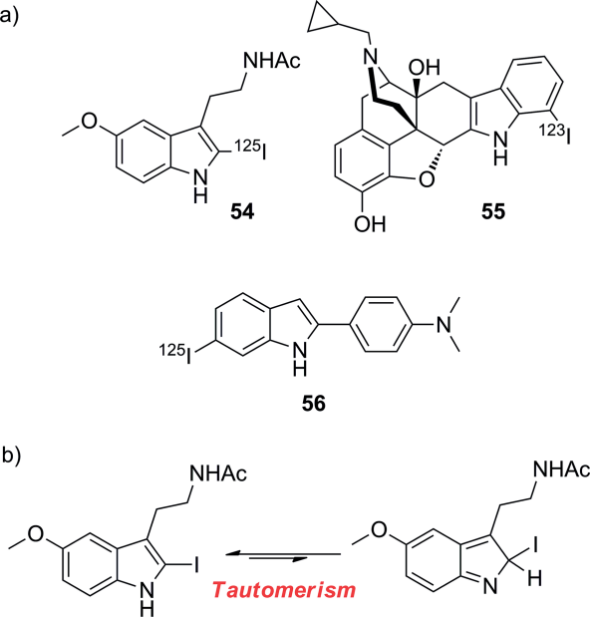
(a) Selected radioiodinated indole compounds. (b) In vivo tautomerism of compound **56**.

Although indoles have been used as sites for labeling with radioiodine, in the synthesis of tracers for in vitro testing, as in the case of the melatonin analogue **54** (Scheme 16a),^81^ they do not find wide use in vivo,^82^ most likely due to their chemical analogy with iodophenols, which makes them particularly prone to in vivo deiodination. In fact, as shown in Scheme 16b, compound **54** may tautomerize (like phenols), facilitating the in vivo removal of iodide. Radioiodinated naltrindole analogue **55**, bearing iodine on C‐5, proved to be stable enough to be used for ex vivo brain autoradiography in mice.^83^ However, in view of the fact that free radioiodide is actively pumped out of the brain, deiodination of iodonaltrindole **55** might have escaped detection, because the autoradiographic analysis was performed exclusively on the brain. The same can be stated for compound **56**, which is a candidate for in vivo imaging of β‐amyloid,^84^ for which deiodination might be considered a negligible side‐reaction, while not hampering the analysis in the brain, if it is assumed that deiodination is slow enough to allow accumulation of the intact pharmaceutical to a detectable concentration.

#### Imidazoles

**Scheme 17:**
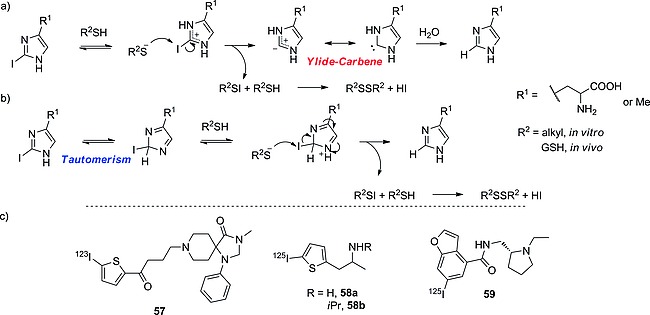
Mechanisms proposed by Goldberg and co‐workers for the in vivo deiodination of iodohistidine: (a) through the formation of an ylide‐carbene leaving group, and (b) through tautomerism. (c) Selected compounds bearing radioiodine on thiophene and benzofuran moieties.

2‐Iodinated imidazoles, as proposed by Goldberg and co‐workers, are biologically not stable, due to the decrease in the BDE of the C–I bond upon protonation.^85^ Goldberg's group proposed two mechanisms for deiodination: in the mechanism depicted in Scheme 17a the driving force would be the formation of an ylide‐carbene leaving group, which should allow the nucleophilic attack of a thiolate (present in vivo in cysteine‐dependent proteins, as GSH) to the iodine atom, whereas in Scheme 17b tautomerism would instead be the kinetically limiting step, as seen before. Goldberg envisaged the mechanism in Scheme 17a, because they observed that methylation of N‐3 was not preventing the reaction from occurring. Nevertheless, they observed that an EWG at C‐4 could hinder the deiodination, whereas the increase in basicity of the imidazole ring should favor it, leaving this mechanism still not completely understood.

#### Benzofuran, Thiophene

Iodo‐substituted thiophenes are biologically more stable than their imidazole counterparts, as in the case of compound **57** (Scheme 17c)^86^ but still seem to have the tendency to deiodinate quite quickly, as can be seen with compounds **58a** and **58b**,^87^ shown in Table 12. Compounds **58a** and **58b** may undergo enzymatic deiodination through the action of IYD, because they share some structural similarities with iodotyramine. Furthermore, their oxidative metabolism may lead to the formation of metabolites that may deiodinate very rapidly. Oxidative metabolism also could explain why compound **57** is more resistant to dehalogenation than **58ab**. In fact, **57** is much larger than **58ab**, and oxidation is likely to occur on parts of the molecule other than the iodothiophene moiety, avoiding the formation of deiodinating metabolites. With the same reasoning the improved biostability of the analogue **58b** relative to that of analogue **58a** could also be explained. Analogue **58b** carries a bulky *i*Pr group, which is also likely to hinder the metabolic enzymes sterically.

**Table 12:** Accumulation of iodine radioactivity in the thyroid for compounds **57**–**59**

	Unit	5 min	10 min	0.5 h	45 min	1 h	2 h	24 h	Ref.
**57** a	ID/g %	0.12 ± 0.06	0.66 ± 0.32	1.51 ± 0.42^d^	2.88 ± 1.00	3.96 ± 0.87	9.17 ± 5.94		86
**58a** b	ID/g %	15.57		50.58		109.68			87
		(13.20–17.31)		(37.97–75.23)		(81.37–166.09)			
	ID/organ %	0.19		0.57		2.25			87
		(0.16–0.20)		(0.41–0.83)		(2.16–2.39)			
**58b** b	ID/g %	12.51		26.26		36.45			87
		(7.87–16.51)		(5.03–36.88)		(31.90–39.98)			
	ID/organ %	0.13		0.28		1.24			87
		(0.08–0.17)		(0.05–0.38)		(0.99–1.40)			
**59** c	ID/organ %	0.06	0.03	0.02		0.02	0.08	0.358	88
		(0.05–0.07)	(0.02–0.03)	(0.01–0.02)		(0.01–0.02)	(0.04–0.11)	(0.259–0.422)	

a Rats, *n* = 3.

b Rats, *n* = 2.

c Rats, *n* = 3.

d 20 min.

Iodobenzofuran seems a promising site for the radioiodination of pharmaceuticals for use in vivo: compound **59** (Scheme 17c) is perfectly stable up to 4 h, and radioactivity in the thyroid rises only after a long residence time in the body (24 h),^88^ due to slow accumulation and organification of radioiodine in the thyroid. Despite the promising profile, there are not many literature examples of successful radiotracers based on this moiety.

### 3.3. Vinyl

**Figure 18:**
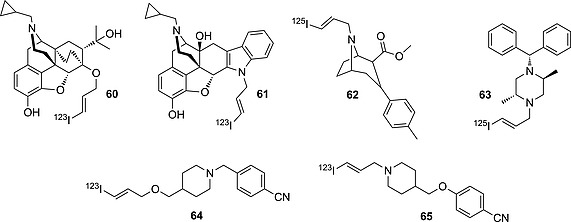
Selected compounds bearing radioiodine on terminal vinyl moieties.

As mentioned before in the Introduction, in the choice of radioiodination site in the design of a radioiodinated tracer, sp^2^ carbon atoms are chosen over sp^3^ ones because of the higher BDE of the C–I bond formed. Also, as seen in the above subsection, some electron‐rich iodoaromatic moieties are well tolerated in vivo. However, in the design of radioiodinated tracers, in cases in which deiodination of an iodoaromatic fragment is of concern, or the iodination results in an analogue that is less potent than the parent compound, the radioactive iodine can be inserted on a vinylic carbon atom, generally on the terminus of an allyl moiety, yielding compounds such as those presented in Figure 18.

The iodoallyl moiety would seem to be particularly convenient in the design of radioiodinated pharmaceuticals, because – in addition to being particularly resistant in vivo, due to the high BDE of the bond formed between the iodine atom and an sp^2^ carbon atom – it can be directly added to a nucleophilic center present in an already known molecule. For instance, compound **60** is a derivative of diprenorphine functionalized with an iodoallyl moiety introduced at a very late stage of the synthesis. Lever and co‐workers claim to have used compound **60** to image peripheral opioid receptors successfully in mice.^83^ The same group anticipated that compound **61**, an iodoallyl derivative of naltrindole, could be used to image Δ‐opioid receptors (DORs) in mouse brain selectively, showing a better pharmacokinetic and pharmacological profile than its iodoaromatic analogue **55**.^90^

As discussed in the Section 2, iodoallyl moieties may undergo oxidation by CYP450 enzymes with consequent loss of the radionuclide, due to the reactivity of the metabolite formed. This effect is more marked for compounds **62**, **63**, and **65** (see Table 13), in which the iodoallyl label is in each case attached to an sp^3^ nitrogen atom. Compound **63** in particular, after very promising in vitro results,^91^ was not selected for further studies due to a poor in vivo profile.^83^ Compound **64**, in which the iodoallyl label is attached to an oxygen atom, proved to be biologically slightly more stable than its analogue **65** (Scheme 16).^78^ The nitrogen atom might activate the oxidative metabolism of the iodoallyl fragment; hence, in the design of new radioiodinated pharmaceuticals bearing such a labeling moiety, an oxygen atom would be preferred over an sp^3^ nitrogen atom as anchoring site.

**Table 13:** Accumulation of iodine radioactivity in the thyroid for compounds **62**, **64**, and **65**

	30 min	1 h	4 h	48 h	Ref.
**62** a	0.27 ± 0.11	n.a.	2.29 ± 0.54	n.a.	89
**64** b	n.a.	0.35 ± 0.14	0.52 ± 0.32	1.99 ± 0.20	78
**65** b	n.a.	0.27 ± 0.14	0.33 ± 0.12	2.34 ± 0.24	78

a ID/organ %, rats, *n* = 5.

b ID/organ %, rats, *n* = 5.

### 3.4. Aliphatic

**Figure 19:**
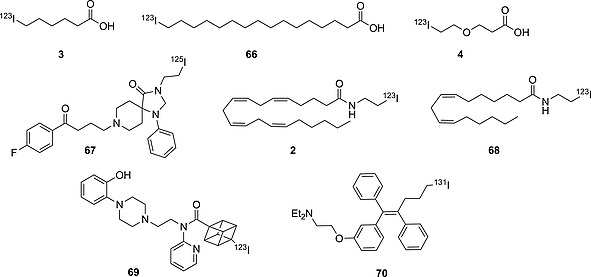
Selected compounds bearing radioiodine on an sp^3^ carbon atom.

Compounds bearing iodine atom on an aliphatic carbon atom are particularly prone to in vivo deiodination, due to the low BDE of the C–I bond. The loss of the radionuclide is likely due to oxidation of the halogenated carbon atom by CYP450 enzymes, as described in Section 2. Radiopharmaceuticals bearing radioactive iodine on an sp^3^ carbon atom are uncommon; however, some research compounds are shown in Figure 19. Compound **3** is rapidly deiodinated (>90 % after 5 min p.i.), whereas its extended analogue **66**, as well as **4**, in which a methylene group at C‐4 is substituted by an oxygen atom, both showed greatly improved biological profiles (>90 % of the ID retained the radionuclide at 5 h p.i.).^25^ Compound **66** may be absorbed and metabolized exactly like a fatty acid and may hide the radioiodinated terminal carbon atom at the center of a micelle, thus avoiding the terminal hydroxylation that would result in deiodination. The γ‐oxygen atom in iodohexanoic acid analogue **4** is likely to block the terminal hydroxylation of the iodinated carbon atom.

Analogously to the β‐iodoethoxy group, the β‐iodoethylamide fragment was successfully used by Chalon's group to obtain the neuroimaging agent **67**, which was only very mildly deiodinated: accumulation of iodine radioactivity in rat thyroid increased from 0.37 (ID/organ %) at 0.5 h p.i. to 2.41 at 5 h.^92^ After this promising result, Wyffels and co‐workers synthesized the anandamide analogues **2** and **68**.^17^ Unfortunately, those compounds were rapidly deiodinated (>45 and >60 %, respectively), as confirmed by ex vivo radio‐HLPC analysis of plasma extractions. Interestingly, in the brain 95 % of the total extracted radioactivity was due to free iodide, meaning either that the deiodination was so fast as to allow iodide to accumulate in brain, or that the deiodination itself occurred in the brain. Most likely compounds **2** and **68**, being very similar analogues of the endogenous neurotransmitter anandamide, underwent metabolic transformations specific to the endogenous substrate directly in the brain, either resulting in loss of the radionuclide or yielding radioactive metabolites that were retained nonspecifically in the brain.

Compound **69** (Figure 19) bears the iodine atom on an unusual cubane polycyclic fragment that proved to be resistant to in vivo deiodination.^93^ The iodocubane is likely not to be oxidized efficiently by liver enzymes, due to its unusual three‐dimensional structure. Also, it is particularly bulky, blocking any possible nucleophilic attack on the halogenated carbon atom. The iodinated tamoxifen analogue **70** (Figure 19), despite bearing the halogen atom on an alkyl chain, turned out to be stable enough in vivo (Table 14) to be used to image expression of estrogen receptors. In fact, compound **70** has several aromatic moieties that are more likely to undergo oxidative metabolism by CYP450 enzymes than the halogenated carbon atom.^94^

**Table 14:** Accumulation of iodine radioactivity in the thyroid for compounds **69** and **70**

	15 min	45 min	1 h	2 h	3 h	6 h	24 h	Ref.
**69** a	0.28 ± 0.01	0.21 ± 0.01	n.a.	0.25 ± 0.03	n.a.	n.a.	n.a.	93
**70** b	n.a.	n.a.	0.393 ± 0.121	0.748 ± 0.076	0.776 ± 0.111	0.696 ± 0.225	0.493 ± 0.122	94

a ID/organ %, rats, *n* = 5.

b ID/g %, rats, *n* = 3.

### 3.5. Summary and Discussion

In this section several radioiodinated tracers have been collected according to the chemical nature of the iodination site. The data on the accumulation of iodine radioactivity in the thyroid have been extracted from biodistribution and pharmacokinetic in vivo studies published in peer‐reviewed journals. Fast accumulation of iodine radioactivity in the thyroid has been taken as a measure of deiodination. The deiodination rate of each pharmaceutical analyzed has been correlated with the specific structural features of the iodination site and of the whole molecule, modeling the biological pathways that those radioiodinated pharmaceuticals were likely to undergo to lead to deiodination. Arenes were defined to be the biologically most stable site for radioiodination. Biostability may be increased through attachment of an electron‐donating substituent (e.g., OCH_3_) to the aromatic ring. Radioiodination on phenols, anilines, heterocycles (excluding benzofuran), and aliphatic carbon atoms yielded, in most cases, biologically unstable structures, which in vivo showed partial or complete loss of the radionuclide. Often, each molecule represents a case to be studied individually, because the whole structure is likely to affect the metabolic pathways the radioiodinated pharmaceutical might undergo. Alternatively, iodoallyl fragments have been successfully incorporated into already known drugs, yielding biologically stable labeled analogues that could be used for ex vivo autoradiography or in vivo imaging.

## 4. Radioiodinated Peptides and Proteins

Recently, interest in radioiodine‐labeled peptides and antibodies has increased. Their high specificity and low toxicity profiles make them useful tools in nuclear medicine.

The iodine radionuclide is commonly incorporated into a peptide chain either through direct iodination of an aromatic moiety or by indirect chemical ligation with an iodinated prosthetic group. In the case of direct iodination, phenylalanine is hard to deiodinate because of the highly electron‐rich character of its aromatic ring. The sulfenyl iodide moiety, formed by radioiodination of cysteine, undergoes fast non‐enzymatic deiodination. Radioiodination of tryptophan is not carried out, not only because of the biological instability of the iodoindole derivative, as discussed in the previous section, but also because during oxidative iodination it undergoes hydrolysis and side‐oxidation reactions resulting in the formation of an unstable oxindole.^95^ Radioiodinated tyrosine and histidine are more stable; of these, tyrosine is preferred because of the milder conditions needed to introduce the iodine and its stability towards non‐enzymatic deiodination.^96^ Radioiodinated aliphatic amino acids are hardly applied because of the low bond dissociation energy (BDE) of the C–I bond formed with an sp^3^ carbon atom. Lysine, arginine, glutamate, and aspartate residues are not commonly radioiodinated; however, their side chains can be used as anchor points to introduce prosthetic groups carrying the radionuclide. Serine and threonine are not as nucleophilic as lysine, and conjugation onto those fragments with a radioiodinated label would need reaction conditions unsuitable for delicate macromolecules such as antibodies. Some artificial amino acids incorporating radioiodine on ad hoc designed lateral chains have been developed (generally iodine is substituted on a vinylic carbon atom), but they are deployed individually as tracers or therapeutic agents in oncology, rather than being incorporated in peptides.^97^

In the following subsections the most important findings in the design of radioiodinated peptides for in vivo studies are reviewed: the different strategies that medicinal chemists have developed to overcome in vivo deiodination of radioiodinated peptides are outlined, and examples of successfully radioiodinated peptides and radioiodinated tracers are given. It is important to note that, apart from the radioiodination site, the pharmacology of the whole labeled peptide may also affect the deiodination rate.

### 4.1. Radioiodine on Tyrosine Moieties

**Figure 20:**
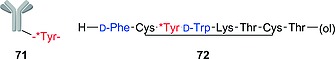
Selected strategies for radiolabeling a peptide by installing radioiodine on a Tyr moiety.

**Scheme 18:**
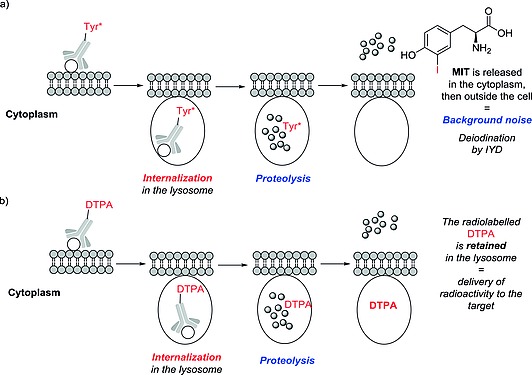
(a) Internalization of a monoclonal antibody radiolabeled by conventional techniques. (b) Internalization of a monoclonal antibody radiolabeled by the technique developed by Stein and co‐workers.

As described by Geissler,^98^ Bakker,^99^ and Foulon,^100^ peptides incorporating radioiodinated tyrosine residues are not susceptible per se to enzymatic deiodination. For instance, Geissler's group came across the first evidence of this when they synthesized the Tyr ^125^I‐labeled monoclonal antibody DA4‐4 (**71**; Figure 20). This particular antibody, having reached its target, was internalized, and after internalization, mostly non‐target signals were observed. The mechanism is depicted in Scheme 18a. This research group discovered that the cellular decrease in radioactivity over time was mostly related to proteolytic catabolism in the internal lysosomes upon internalization of the antibody–target complex.^98^ Enzymatic deiodination could take place only once the iodo‐Tyr moieties had been released as single free amino acid units. Similar findings were obtained by Bakker on determining the pharmacokinetic/pharmacodynamic profile of the radiolabeled ^123^I‐Tyr3‐octreotide **72** (Figure 20). This cyclic peptide contains two d‐amino acids, making it more resistant to proteolysis.[99b] Metabolic studies by the same research group showed that most of the peptide was excreted intact, and the residual non‐specific radioactivity was mostly represented by the drug itself, which was undergoing normal clearance by the liver and the kidneys.[99a] Abnormal radioactivity in the thyroid could be observed only at 24–48 h p.i., meaning that the deiodination process was taking place after a slow proteolytic catabolism.

**Figure 21:**
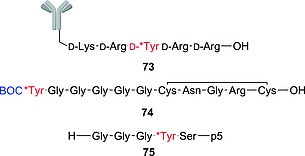
Selected strategies for radiolabeling a peptide by installing radioiodine on a Tyr moiety.

Foulon and co‐workers designed a terminal pentapeptidic group containing a ^131^I‐d‐tyrosine residue and consisting of d‐amino acids, which was fused to an anti‐EGFRvIII monoclonal antibody, yielding the antibody derivative **73** (Figure 21).^100^ The rationale behind their design is that proteolytic metabolism of an antibody bound to its target cannot be prevented, thus at least the radioiodinated label has to be stable towards proteolysis and has to be retained in the lysosomes. The radioiodinated labels NH_2_–KR*YRR–OH and NH_2_–D‐KR*YRR–OH each contained three positively charged amino acids, because previous works^101^ had suggested that positively charged metabolites were retained much longer in the lysosomes without being released in the cytoplasm. The use of d‐amino acids further prevented proteolysis, as was demonstrated by the fact that when tested in vivo, most of the labeled peptide **73** was excreted intact. Thus, the combined effect of the major retention in the lysosome (resulting from the positively charged amino acids) and the improved proteolytic stability (conferred by the incorporation of d‐amino acids) resulted in an improved signal‐to‐noise profile compared to previous efforts with different radioiodinated labels, enabling successful imaging of EGFRvIII by full‐body SPECT.

While designing smaller peptides for use directly as tracers, Sun and co‐workers^102^ discovered that the *t*‐Boc‐protected YG5 peptide **74** (Figure 21) was markedly more stable towards deiodination than its unprotected analogue. The particular feature of this peptide is that the radiolabeled tyrosine residue is terminally located, spaced by five glycine moieties from the pharmacophore (the RGD sequence). This terminal position means that the labeled tyrosine residue is exposed to dehalogenases or proteases, but introducing the *t*‐Boc group on the amine terminus of YG5, as in **74**, likely prevented the peptide from being metabolized, allowing it to be used as a radiotracer.

Wall and his research group assayed a peptide p5 labeled with a radioiodinated tyrosine residue near the N‐terminus, but not as the last amino acid, in compound **75** (Figure 21).^103^ Peptide p5 is not an antibody – it is much smaller – but is believed to bind strongly to its target (amyloid plaques) in a similar fashion. The complex of p5 with the amyloid plaques does not undergo proteolysis (i.e., is not internalized), due to the intrinsic resistance of amyloid plaques to proteolysis, as opposed to unbound **75**, which undergoes fast body clearance. Recording the residual radioactivity of the bound complex allowed use of the peptide as a radiotracer. It is important to mention that the peptide–target complex does not undergo deiodination, thus differing from the fate of the complex formed by the antibody DA4‐4 (**71**) and its target, the lysosomal proteolysis of which, as seen before, is unavoidable.^98^ This observation illustrates the concept that every radioiodinated peptidic tracer makes its own case, needing the medicinal chemist to design each probe according to the fate that the peptide and the possible complex between the peptide and its target are likely to meet.

**Figure 22:**
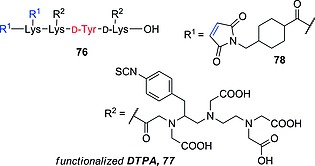
Strategy for radiolabeling a peptide by installing radioiodine on a functionalized Tyr‐containing moiety as described by Stein and co‐workers.^104^

Stein and co‐workers developed a labeling strategy to circumvent the problem of release of radioiodide from the target cell after internalization and digestion of the radioiodinated antibody in the lysosomes.^104^ This labeling approach is shown in Figure 22 and features a label (peptide **76**) to be conjugated to the antibody. Compound **76** is a tetrapeptide containing the two functionalized DTPA prosthetic groups **77** and the two maleimide moieties **78**. The DTPA moieties, attached to the ε‐amino branch of two d‐lysine residues, facilitate the retention of the radiolabel in the lysosome: they each carry several carboxylic acid functionalities, which are known to react in the lysosome with nucleophilic fragments (mechanism depicted in Scheme 18b). The d‐amino acids in the tripeptide prevent the radiolabel being cleaved from the DTPA moiety, and as a result the radioactive catabolites are retained in the lysosomes of the target cell. The *para*‐NCS‐benzyl group was introduced to adjust the hydrophobicity of the molecule in order to improve the uptake into tumor cells. Radioiodine was installed onto the d‐tyrosine moiety (red in Figure 22) and onto the maleimido moieties (double bond highlighted blue in Figure 22). The latter were introduced ad hoc to improve the radioiodination yield.

This probe represents the current state of the art for labeling antibodies with a residualizing radioiodine label. Tumor‐to‐blood and tumor‐to‐muscle ratios were 10 times higher when the radiolabeling strategy of Stein and co‐workers was applied than they were when the same antibody was labeled by conventional radioiodination techniques (i.e., chloramine‐T method).

### 4.2. Radioiodine on Prosthetic Groups

**Scheme 19:**
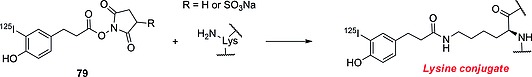
Formation of the lysine conjugate with the Bolton–Hunter reagent **79**.

The cases analyzed until now mostly relate to the radioiodination of a tyrosine moiety. In the literature, though, there are several examples based on prosthetic groups, commonly an iodinated aromatic acyl moiety, attached to a nucleophilic lateral chain, such as through the nitrogen atom in lysine (Scheme 19). Obviously, the main drawback of this method is that the acylation site must not involve its pharmacophore. It is mostly exploited in the labeling of longer peptides or monoclonal antibodies, because these are large globular proteins that can tolerate modification, as long as the prosthetic groups are conjugated far away from the recognition epitopes.

One of the first, and most widely used, methods to perform peptide labeling of lysine is the conjugation with a Bolton–Hunter reagent of type **79** (Scheme 19).^105^ Russell and co‐workers compared the use of the Bolton–Hunter reagents with the iodination carried out on nonspecific tyrosine moieties, and found the Bolton–Hunter reagent conjugates to be more resistant to deiodination than the tyrosine labeling products.^106^ Although they did not further investigate the factors affecting deiodination, nor the effect of the position in the peptide in which modification was occurring, it can be assumed that the lysine conjugate (Scheme 19) does not undergo proteolytic cleavage, preventing the release of radioiodinated Bolton–Hunter label. However, the nonconjugated Bolton–Hunter reagents share many of structural features with 4‐iodotyrosine, being very likely good substrates for deiodinases.

**Figure 23:**
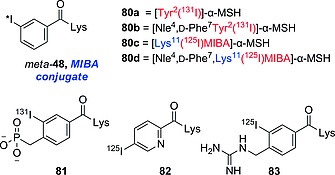
Selected lysine conjugates formed with derivatives of iodobenzoic acid **48**.

In the course of defining the scope of different labeling methods for peptides, Garg's group compared the biostability towards deiodination of peptides **80c** and **80d** (Figure 23), labeled with *meta*‐**48** (MIBA), with that of peptides **80a** and **80b** (Figure 23), in which iodine was introduced directly on an iodotyrosyl moiety. Modifications in the nearby amino acid sequence were then introduced, to investigate whether the neighboring amino acids would affect the deiodination rate of the iodine‐bearing label.^107^ These modifications consisted of changing a methionine residue for a norleucine one and the insertion of a d‐amino acid (**80a** and **80c** are the parent peptides; **80b** and **80d** are their modified analogues). The methionine modification was assayed to investigate the hypothesis of a neighboring deiodination effect by a nearby thiol moiety, whereas the d‐amino acid was inserted to improve the proteolytic stability of the modified analogues. For the two tyrosine‐labeled peptides **80a** and **80b**, fast accumulation of radioiodide in the thyroid was observed, whereas the two peptides **80c** and **80d**, labeled with *meta*‐**48**, gave superb signal‐to‐noise ratios. Radioiodide accumulation in the thyroid could be observed only a long time after injection. The main radioactive metabolite of **80a** and **80b** was reported to be free radioiodide, whereas for peptides **80c** and **80d** the radioactivity was mostly excreted as lysine labeled with MIBA. No significant difference between the altered‐sequence peptides was observed, probably because the neighboring deiodinating effect by the thiol moieties might have been observed only when placed exactly next to the iodine label. In addition, the d‐amino acids were introduced quite far from the iodinated moiety, which did not prevent proteolysis of the label.

Conjugates **81**–**83** (Figure 23) are lysine derivatives labeled with aromatic acyl moieties, which are charged at physiological pH.[101a], 108 Conjugate **81** proved to be negatively charged even at lysosomal pH, whereas **82** and **83** are positively charged. No important accumulation in the thyroid was observed after injection of the bioconjugate derivatives **81**–**83**, making them good candidates for use in clinical radiology. The biostability towards deiodination is most likely given by the capability of the radioiodinated label to be retained in the lysosome, being charged at lysosomal pH. Nevertheless, because the acylation is likely to happen on the most exposed fragments on the surface of the protein, the three‐dimensional structure may isolate the labeled fragments from the surrounding matrix, avoiding contact with the solvent or free fragments that may interact with the radioiodinated moiety.

### 4.3. Summary and Discussion

The results reviewed in this section indicate that proteolysis plays a major role – even more important than that played by deiodinases – in the deiodinating catabolism of radioiodinated peptides and proteins. It is an aspect that is still not completely understood and one that would need further studies. It is, however, possible to define a few rules for the design of radioiodinated peptidic tracers.

Iodination on tyrosine moieties is the most effective peptide labeling technique, because it involves a non‐peptidic modification that would normally not affect the conformation of the pharmacophore. However, if the labeled tyrosine residue and its iodination state are essential to the pharmacophore, another labeling technique must be identified. It is important to know the fate of the peptide once it reaches its target: if the complex is likely to undergo internalization and subsequent proteolysis in the lysosomes, it is crucial either to prevent the proteolysis or to force the catabolites not to be released in the cytoplasm. Proteolysis can be avoided by incorporating the Tyr in a peptidomimetic feature such as inside a cyclic structure (as in the case of compound **72**) or among non‐proteinogenic amino acids (compound **73**). Monoclonal antibodies, which are internalized upon reaching their target, can instead be conjugated to a radioiodinated label, which – upon internalization and subsequent degradation – is not released in the cytoplasm as radioactive catabolites, being designed to be retained in the lysosome (as in the cases of labels **73**, **76**, **81**, **82**, and **83**). If this is not possible, because it would affect the pharmacology of the probe, an iodinated tyrosine unit can be attached to a polyglycine spacer terminally protected with *t*‐Boc (as in the case of compound **74**) to prevent proteolysis. If none of those modifications are tolerated or it is not possible to introduce them efficiently, the use of a prosthetic group bearing the radionuclide can be envisaged (as in the cases of labels **79** and *meta*‐**48**). When the peptide–target complex is not subject to proteolytic degradation, simple radioiodination of tyrosine fragments (as in radiolabeled peptide **75**) will be more useful, because the unbound probes will be cleared out, removing non‐target signals. Finally, it is important to avoid directly neighboring thiol‐containing amino acids, because they would intramolecularly remove the iodine as mentioned in Section 2. Furthermore, there is evidence that the use of radioiodinated metabolically stable tyrosine analogues does not yield antibody conjugates that can be successfully used as tracers, because upon proteolytic cleavage the free amino acid would diffuse non‐specifically to non‐target tissues, lowering the signal‐to‐noise ratio in the analysis.^53^

This section reviews some of the most important and recent advances in the design of radiolabeled peptides and proteins with radioactive iodine. Different strategies (direct iodination and prosthetic‐group conjugation) are discussed with particular focus on the factors that may affect the dissociation of the radioiodine from the tracer. The main factor involved is identified as the proteolytic cleavage of the radioiodine label; no on‐peptide deiodination is observed. Thus, some strategies to improve the surrounding of the radioiodine to improve the metabolic stability are discussed. Finally, it has become clear that the strategy to be deployed has to be adapted to each particular drug, because – to design the appropriate probe – it is necessary to understand the metabolic fate of the whole radiolabeled drug. Lastly, further studies might be directed towards clear understanding of the role of deiodinases and proteolytic cleavage during the in vivo deiodination of radiolabeled peptides.

## 5. General Guidelines

A number of factors need to be taken into account in the design of radioiodinated pharmaceuticals to improve biostability towards in vivo deiodination.

The deiodination rate is determined by the physiochemical properties of the whole molecule. In this review we have also aimed to identify the impact of the physiochemical properties of the iodination site. In Subsection 5.1 we review the factors relating to the composition of the whole molecule that may affect the deiodination rate. In Subsection 5.2, the structural and chemical composition of the radioiodination sites and the correlation with the rate of deiodination are analyzed. On the basis of this analysis, the radioiodination sites are classified as resistant or not resistant towards in vivo deiodination. Finally, an example of design strategy is discussed, with the derived guidelines and the knowledge gathered in this review being applied in the rational design of a radioiodinated pharmaceutical with improved biostability towards in vivo deiodination.

### 5.1. Analysis of the Whole Molecule

It is crucial to understand how the whole molecule is metabolized, because metabolic transformations of other groups may affect the deiodination rate, even though they would not appear to affect the reactivity of the C–I bond directly. Generally, xenobiotics are metabolized to less lipophilic derivatives to facilitate their excretion through the kidneys. Metabolic transformations on the same moiety as used for radioiodination are likely to result in cleavage of the C–I bond, either through direct deiodination (as in the case of deiodination of iodotyramine‐/iodothyronine‐like molecules catalyzed by DIOs/IYD and of activated *para*‐iodoanilines by CYP450 enzymes, as shown in Section 2), or through formation of deiodinating metabolites (as in the cases of oxidation of alkyl and vinyl iodides, activated *ortho*‐iodoanilines, or compound *meta*‐**47**, as shown in Subsections 2.3 and 2.4 and in Scheme 15b in Subsection 3.1, respectively). When a metabolic transformation is not directed at the radioiodination site, two main kinds of metabolites can be defined: deiodinating and non‐deiodinating.

Deiodinating metabolites are radioiodinated metabolites that either dehalogenate spontaneously under physiological conditions (e.g., α‐hydroxy halides such as **14** in Scheme 10) or are substrates for deiodinating enzymes (e.g., MIBG metabolite **50** in Scheme 15b, *ortho*‐iodoanilines as shown in Scheme 14, or peptides such as **71** in Figure 20, which release MIT upon proteolytic catabolism).

Non‐deiodinating metabolites are radioiodinated compounds that have been transformed into less lipophilic metabolites without the physicochemical properties of the C–I bond being affected; they are less likely to be further metabolized at the radioiodination site, avoiding further deiodination.

**Figure 24:**
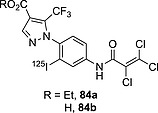
Compounds tested by Hagimori and co‐workers.^109^

Compound **84a** (Figure 24), for instance, was deiodinated twice as rapidly as its metabolite **84b**, as shown in the biodistribution study by Hagimori and co‐workers reported in Table 15, in which **84a** and **84b** were tested separately.^109^ The deiodination of compound **84a** likely involved oxidative metabolism, because it was importantly accumulated early p.i. in the liver and in the lung (tissues rich in CYP450 enzymes), whereas compound **84b** was accumulated at a much slower rate. Hagimori and co‐workers also observed that compound **84a** was metabolized in the serum to **84b**, so the higher accumulation of the radioiodine in the thyroid may be related to early deiodination of the parent compound **84a**, before it was completely hydrolyzed in the serum to **84b**. Hypothetically, inhibiting the hydrolysis of the ethyl ester in the serum, or replacing the ethyl ester with a non‐hydrolyzable lipophilic bioisostere (e.g. 3‐ethyl‐1,2,4‐oxadiazole), could result in an analogue less resistant than compound **84a**, because the analogue would undergo more oxidative metabolism, also of the radioiodinated site.

**Table 15:** Accumulation of iodine radioactivity in organs of interest (mice) for compounds **84a** and **84b**, *n* = 5, as described by Hagimori and co‐workers^109^

	Organ	10 min	0.5 h	1 h	3 h	24 h
**84a**	thyroid^a^	0.13 ± 0.03	0.14 ± 0.03	0.16 ± 0.05	0.19 ± 0.08	0.29 ± 0.11
	lung^b^	20.58 ± 2.64	5.65 ± 1.12	4.68 ± 0.78	1.61 ± 0.23	0.66 ± 0.10
	liver^b^	14.59 ± 2.91	6.16 ± 2.57	7.80 ± 2.64	4.29 ± 1.51	0.21 ± 0.03
**84b**	thyroid^a^	0.10 ± 0.02	0.10 ± 0.01	0.10 ± 0.01	0.13 ± 0.02	0.13 ± 0.03
	lung^b^	1.31 ± 1.02	0.76 ± 0.40	0.59 ± 0.03	0.57 ± 0.10	0.45 ± 0.07
	liver^b^	12.22 ± 2.60	9.37 ± 1.87	8.94 ± 6.27	8.08 ± 2.75	0.12 ± 0.02

a ID/organ %.

b ID/g %.

The metabolic profile of the non‐iodinated analogue of the radioiodinated pharmaceutical of interest may give useful information for directing radioiodination to a site not already known to be oxidized by CYP450 enzymes or generally not modified in vivo. If another moiety in the molecule of interest, far from the radioiodination site, is known to be metabolized, it is likely that the metabolite will be more resistant than the parent compound (e.g., compound **84a**). More broadly, decreasing the C log P value for the system of interest (i.e., obtaining less lipophilic analogues, as shown for estradiol analogue **20a** in Subsection 2.3, Figure 7, or in the text above for compound **84b**), may reduce the extent of metabolism by oxidative deiodination, yielding more resistant analogues. If the pharmacology of the compound does not allow less lipophilic analogues, the presence of other apolar moieties that are better substrates than the radioiodinated carbon atom for oxidation by CYP450 enzymes may decrease the oxidative deiodination (as shown in Subsection 3.4 for tamoxifen analogue **70** (Figure 19) the three aryl moieties of which are more likely to be oxidized than an iodinated sp^3^ carbon atom, or for compound **4** (Figure 2) in which the γ‐oxygen atom makes β‐oxidation of the carboxylic acid more likely than the deiodinating hydroxylation of the iodinated carbon atom). Furthermore, steric hindrance and bulk around the iodination site were found to yield more resistant radioiodinated pharmaceuticals, because the radioiodinated moiety is less accessible to the metabolic enzymes (as in the case of compound **69**). When deiodination is carried out by DIOs or IYD enzymes, the use of more constrained scaffolds yields more resistant radioiodinated pharmaceuticals (as in the case of morphine analogue **31**). The specific pharmacology and pharmacokinetic profile of the radioiodinated pharmaceutical may affect the deiodination rate, as observed in Subsection 3.4 for compound **66**. This does not substantially differ from naturally occurring fatty acids and is likely metabolized as such. Compound **66**, once injected, is likely to be transported in the body in micelles, with the terminal halide hidden at the center. Hence, the β‐oxidation of the carboxylic polar head (as a naturally occurring fatty acid) is more likely to occur rather than the hydroxylation of the hidden terminal iodinated carbon atom.

Also, it is important to consider the fate of the radioiodinated pharmaceutical. For instance, radioiodinated peptides that undergo internalization and proteolysis by the target cell can be radioiodinated through specifically designed moieties that will be retained in the target, avoiding the release of deiodinating metabolites.

Radioiodinated pharmaceuticals acting in the central nervous system, crossing the BBB, might not be hindered by deiodination unless the deiodination rate outside the brain is faster than the pharmacokinetics of the compound (i.e., no radioactive compound can reach its target in the brain), or the deiodination takes place inside the brain (i.e., iodide is not cleared rapidly enough from the brain, hampering the analysis).

### 5.2. Analysis of the Radioiodination Site

The structural features of the iodinated radiopharmaceuticals covered in this review are collected here, and their resistance or lack of resistance towards in vivo deiodination is briefly resumed.

Radioiodinated sp^2^ carbon atoms often yield C–I bonds that are more resistant towards in vivo deiodination than sp or sp^3^ carbon atoms.

Iodoarenes and iodovinyl systems are generally resistant towards in vivo deiodination.

*meta*‐Iodoarenes are generally more resistant than their *ortho* or *para* isomers towards in vivo deiodination.

Iodoanilines are often not resistant towards in vivo deiodination. *meta*‐Iodoanilines are more resistant than their *ortho* or *para* isomers.

Iodophenols, especially *ortho*‐iodophenols, are often not resistant to in vivo deiodination. Non‐strained and non‐bulky iodotyramines in particular are not resistant towards in vivo deiodination.

Electron‐donating groups (e.g. methoxy, methyl, …) on iodoarenes generally yield compounds resistant towards in vivo deiodination.

Electron‐withdrawing groups (e.g., nitro, carboxyl, …) on iodoarenes generally yield compounds not resistant towards in vivo deiodination.

Difluorination in the vicinity of the radioiodine generally yields iodoarenes that are more resistant towards in vivo deiodination.

Iodovinyl moieties are often deployed in the form of iodoallyl fragments used to radioiodinate a nucleophilic oxygen or nitrogen atom. When an oxygen rather than a nitrogen atom has been iodoallylated, the resulting compounds have generally been more resistant towards in vivo deiodination.

Radioiodinated heterocycles are often not resistant to in vivo deiodination. Radioiodinated nitrogen‐containing (e.g., quinozalines, indoles, or imidazoles) and sulfur‐containing (e.g., thiophenes) heterocycles are generally not resistant towards in vivo deiodination. Radioiodinated oxygen‐containing heterocycles (e.g., benzofurans) are one of the few classes of iodinated heterocyclic moieties to be resistant to in vivo deiodination.

Radioiodination on sp^3^ carbon atoms should yield radioiodinated compounds that are resistant towards in vivo deiodination when: (i) other, more accessible lipophilic fragments are present, (ii) the sp^3^ carbon atom is embedded in a bulky (e.g., cubane) moiety, or (iii) the radioiodine is inserted in the form of a 2‐iodoethoxy or (2‐iodoethyl)amino moiety.

### 5.3. Example of Rational Design, Design of Radioiodinated THC Analogues

Here the derived guidelines and the knowledge gathered in this review are applied to the rational design of iodinated pharmaceuticals with improved in vivo biostability towards deiodination. The following molecules were chosen, because they are abundant natural products [i.e., complex molecules that can easily be obtained and functionalized (radioiodinated)]. Also, the corresponding “cold” iodinated analogues of those natural products are already known and have been pharmacologically tested, allowing us to rationally infer why no “hot” analogue has ever been tested in vivo and which rational modification we would apply to improve the in vivo profiles of such radiopharmaceuticals.

**Scheme 20:**
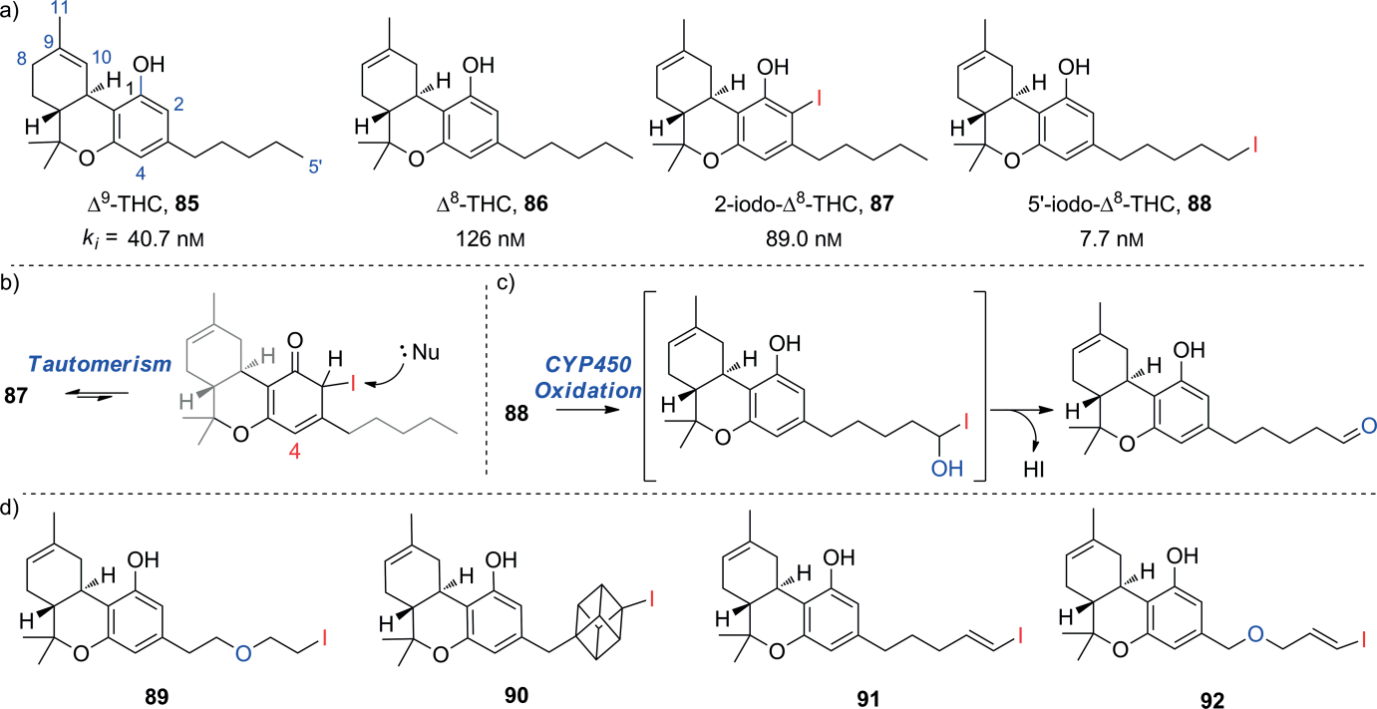
(a) Selected cannabinoid analogues. (b) Tautomerism of analogue **87**. (c) Proposed mechanism for the in vivo deiodination of compound **88**. (d) Iodinated analogues of compound **86** that might show improved in vivo profiles in relation to compounds **87** and **88**.

Compound **85**, Δ^9^‐tetrahydrocannabinol (Δ^9^‐THC; Scheme 20a), is the principal psychoactive ingredient in *Cannabis sativa*. The recent difficulties encountered by Bial Pharmaceutics in developing pharmaceuticals targeting the endocannabinoid system^110^ shed light on the lack of knowledge relating to the differences in pharmacology and pharmacokinetics between synthetic and natural occurring cannabinoids. A short (1 h) biodistribution study, deploying a 5′‐radiofluorinated analogue of **86**, through PET and plasma radioactivity count, was successfully performed by Charalambous and co‐workers.^111^ Notably, they could observe evidence of defluorination of their analogue. No radioiodinated analogues of **85** or **86** could successfully be deployed to image the cannabinoid system, making it difficult to study the long‐lasting pharmacological effects of those naturally occurring compounds. “Cold” iodinated analogues in the form of compounds **87** and **88** were synthesized and tested, both in vitro and in vivo, in a SAR (structure–activity relationship) study by Compton and co‐workers.^112^ Structural modifications geared towards the study of the pharmacological activity of **85** have often been performed on a cannabinoid scaffold such as compound **86**, which has pharmacological effects comparable with those of **85**, but was reported to be chemically more stable.^113^ No imaging studies deploying radioiodinated analogues of THC have been published to date, presumably due to poor in vivo profiles of radioiodinated compounds **87** and **88** as tracers, which may have been detected in pilot studies. The poor in vivo profiles could be explained by in vivo metabolic deiodination, which can be elucidated and overcome by using the guidelines outlined above.

In compound **87** (Scheme 20a), the iodine atom is installed on a substituted arene, an *ortho*‐iodophenol, a structure that has been seen before (Subsection 3.2.2) and shown to have potential to deiodinate. In view of the bulky strained three‐dimensional structure of compound **87**, and the lack of ionizable anchor points, it is unlikely that it might be a substrate for deiodinases, so a supposed poor in vivo profile could be a consequence of interaction between nucleophilic moieties (e.g., the thiol terminus of a cysteine) and the reactive tautomer of compound **87** (shown in Scheme 20b). Shifting the position of the iodine atom on the aromatic ring (from 2 to 4) might help to circumvent the deiodination side‐reaction; however, substitution at the 4‐position of THC is already known to suppress any bioactivity.^113^ For this reason 4‐substitution with an electron‐donating group is not useful either; therefore, to design a radioiodinated analogue of compound **86** that may be biologically stable, another site for radioiodination must be chosen.

In compound **88** (Scheme 20a) the iodine atom was installed on a terminal sp^3^ carbon atom of a linear pentyl moiety, a structure that has been seen before (Subsection 2.3.2) and has the potential to undergo oxidative deiodinating metabolism by CYP450 enzymes. These would oxidize the 5′‐position, resulting in a very unstable α‐hydroxy halide, which would quickly deiodinate to yield the corresponding aldehyde (Scheme 20c). Compound **85** is known also to undergo oxidative metabolism at the 7‐, 8‐, 9‐, 10‐, and 11‐positions in humans, as shown by Watanabe and co‐workers;^114^ therefore, radioiodination at those positions could also yield analogues likely to show poor in vivo profiles, due to deiodination by oxidative metabolism. Deiodination originating from oxidation by CYP450 enzymes at the 5′‐position can be circumvented by deploying the strategies shown above (Subsection 3.5): that is, by installing a β‐iodoethoxy group, as in analogue **89** (Scheme 20d), or iodine on a bulky three‐dimensional structure (e.g., a cubane), as in analogue **90** (Scheme 20d). Alternatively, radioiodine can be installed through an sp^2^ carbon atom in a terminal vinyl or allyl moiety, as shown before (Subsection 3.4), generating analogues such as **91** or **92** (Scheme 20d).

Analogues **89**–**92**, generated according to the guidelines in this review, could potentially be deployed as SPECT or PET in vivo tracers because of their hypothesized improved resistance towards in vivo deiodination, in relation to the already known iodinated compounds **87** and **88**. The use of **89**–**92** could allow the study of the long‐term interactions of the natural occurring compounds such as **85** and **86** with the endocannabinoid system through in vivo imaging.

### 5.4. Synthesis of Radioiodinated Pharmaceuticals

**Scheme 21:**
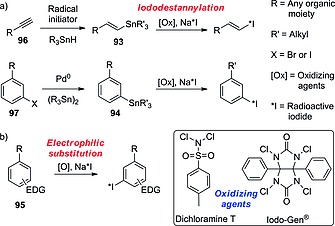
Commonly used reactions and reagents for the radioiodination of pharmaceuticals.

Because radioiodination reactions of pharmaceuticals have been extensively reviewed elsewhere,^115^ here we only briefly summarize the techniques most commonly used to insert radioiodine onto the scaffolds considered in this review to be most resistant towards in vivo deiodination (i.e., radioiodinated vinylic carbon atoms and electron‐rich arenes). Radioiodine isotopes (*I) are generally purchased as Na*I solutions in aqueous NaOH. The iododestannylation of a trialkyltin precursor (compounds such as **93** and **94** in Scheme 21a) is the preferred synthetic route, allowing iodine to be inserted with high regioselectivity. In the case of particularly strongly activated arenes (compounds such as **95**), electrophilic substitution is also a viable route (Scheme 21b).^115^ In both iododestannylation and electrophilic substitution, *I^+^ is generated in situ from Na*I in the presence of an oxidizing agent, to displace a leaving group (the trialkylstannyl group and a proton, respectively). Commonly used oxidizing agents are hydrogen peroxide, peracetic acid, dichloramine T, and Iodo‐Gen® (Scheme 21, rounded square).^115^ The trialkyltin precursor is often obtained through radical hydrostannylation of an alkyne **96** with R′_3_SnH or through Pd^0^‐catalyzed substitution of a trialkyltin group onto a halogenated arene **97**.
